# A phospho-dawn of protein modification anticipates light onset in the picoeukaryote *Ostreococcus tauri*

**DOI:** 10.1093/jxb/erad290

**Published:** 2023-07-22

**Authors:** Zeenat B Noordally, Matthew M Hindle, Sarah F Martin, Daniel D Seaton, T Ian Simpson, Thierry Le Bihan, Andrew J Millar

**Affiliations:** SynthSys and School of Biological Sciences, University of Edinburgh, Edinburgh EH9 3BF, UK; SynthSys and School of Biological Sciences, University of Edinburgh, Edinburgh EH9 3BF, UK; SynthSys and School of Biological Sciences, University of Edinburgh, Edinburgh EH9 3BF, UK; SynthSys and School of Biological Sciences, University of Edinburgh, Edinburgh EH9 3BF, UK; Institute for Adaptive and Neural Computation, School of Informatics, University of Edinburgh, Edinburgh EH8 9AB, UK; SynthSys and School of Biological Sciences, University of Edinburgh, Edinburgh EH9 3BF, UK; SynthSys and School of Biological Sciences, University of Edinburgh, Edinburgh EH9 3BF, UK; University of Glasgow, UK

**Keywords:** Light signalling, marine microalgae, phosphoproteomics, photoperiod, photosynthetic pico-eukaryotes, proteomics, systems biology

## Abstract

Diel regulation of protein levels and protein modification had been less studied than transcript rhythms. Here, we compare transcriptome data under light–dark cycles with partial proteome and phosphoproteome data, assayed using shotgun MS, from the alga *Ostreococcus tauri*, the smallest free-living eukaryote. A total of 10% of quantified proteins but two-thirds of phosphoproteins were rhythmic. Mathematical modelling showed that light-stimulated protein synthesis can account for the observed clustering of protein peaks in the daytime. Prompted by night-peaking and apparently dark-stable proteins, we also tested cultures under prolonged darkness, where the proteome changed less than under the diel cycle. Among the dark-stable proteins were prasinophyte-specific sequences that were also reported to accumulate when *O. tauri* formed lipid droplets. In the phosphoproteome, 39% of rhythmic phospho-sites reached peak levels just before dawn. This anticipatory phosphorylation suggests that a clock-regulated phospho-dawn prepares green cells for daytime functions. Acid-directed and proline-directed protein phosphorylation sites were regulated in antiphase, implicating the clock-related casein kinases 1 and 2 in phase-specific regulation, alternating with the CMGC protein kinase family. Understanding the dynamic phosphoprotein network should be facilitated by the minimal kinome and proteome of *O. tauri*. The data are available from ProteomeXchange, with identifiers PXD001734, PXD001735, and PXD002909.

## Teaser

The phosphorylation of 66% of phosphoproteins was rhythmic under light–dark cycles, and suggested circadian control by particular kinases. The <10% rhythmic protein profiles reflected light-stimulated protein synthesis in this microalga.

## Introduction

Responses to light are critical for organisms of the green lineage (Noordally and Millar, 2015; Paajanen *et al.*, 2021). The rapid effects of photosynthetic light harvesting, for example on redox state and sugar metabolism, are complemented by signalling photoreceptors (Whitelam and Halliday, 2007) and the slower, 24 h regulation by the biological clock (Millar, 2016; Creux and Harmer, 2019). Circadian regulation allows organisms to anticipate the predictable, day–night transitions of the diel cycle, complementing the responses to faster changes in light levels (Troein *et al.*, 2011). Mehta *et al.* (2021) refer to these as ‘anticipatory’ and ‘reactive’ regulation. At the macromolecular level, the transcriptomes in the green lineage show widespread and overlapping regulation of mRNA abundance by both light and circadian signals (see below), whereas the diel regulation of proteins and their post-translational modifications had been less studied (Mehta *et al.*, 2021). We addressed that gap using a minimal biological system, focusing on protein phosphorylation.

Phosphorylation of an existing protein is energetically inexpensive, occurs rapidly, and can then alter protein activity through conformational change or intermolecular recognition (Khoury *et al.*, 2011). These characteristics seem fitted to reactive regulation. Some plant photoreceptor proteins include protein kinases that initiate light signalling (Christie, 2007; Djouani-Tahri el *et al.*, 2011a).

Protein synthesis is not only far slower but also among the costliest macromolecular processes (Scott *et al.*, 2010; Karr *et al.*, 2012), seemingly more suited to anticipatory regulation. Rhythmic regulation might then provide a selective advantage, loosely summarized as making proteins when they are needed in the diel cycle (Laloum and Robinson-Rechavi, 2022). That reasoning helped to interpret the co-regulation of functional clusters of RNAs, when transcriptome studies demonstrated that >50% of Arabidopsis RNAs can be rhythmic under diel, light–dark (LD) cycles (Smith *et al.*, 2004; Blasing *et al.*, 2005; Michael *et al.*, 2008). Most strikingly, almost the whole transcriptome of the marine unicellular alga *Ostreococcus tauri* was rhythmic in controlled conditions (Monnier *et al.*, 2010), and this was also the most rhythmic taxon among the diverse plankton of a Pacific time series (Kolody *et al.*, 2019). The clock might also allow anticipation, to ensure that the proteins had been fully synthesized and assembled to their active state by the appropriate time.

Proteomic data, in contrast, revealed that the detected proteins had stable levels, with an average half-life of >6 d in the model plant *Arabidopsis thaliana* (Li *et al.*, 2017), suggesting little scope for diel rhythmicity. Time series under constant light or a diel cycle found up to 6% of rhythmic proteins (Baerenfaller *et al.*, 2012, 2015; Choudhary *et al.*, 2016; Uhrig *et al.*, 2021; Krahmer *et al.*, 2022). The shortest lived, regulatory proteins are harder to detect, but such proteins seem to be exceptions to the general protein stability, consistent with mammalian systems (Doherty *et al.*, 2009). Global regulation of protein synthesis is also clearly relevant in plants and algae (Piques *et al.*, 2009; Juntawong and Bailey-Serres, 2012; Pal *et al.*, 2013; Ishihara *et al.*, 2015; Missra *et al.*, 2015). In this context, circadian RNA regulation was proposed to offer a selective advantage through seasonal adaptation of protein levels to day-length on a time scale of weeks (Seaton *et al.*, 2018).

More protein phosphorylation sites change over the diel cycle, compared with protein levels (Kusakina and Dodd, 2012; Mehta *et al.*, 2021). Protein phosphorylation in plants and algae is most directly light regulated by the photoreceptor kinases (Christie, 2007; Djouani-Tahri el *et al.*, 2011a), though light also affects the broader phosphoproteome (Turkina *et al.*, 2006; Boex-Fontvieille *et al.*, 2014; Schönberg *et al.*, 2017), for example affecting 25% of Arabidopsis phosphopeptides within 30 min (Uhrig *et al.*, 2021). Circadian studies in Arabidopsis under constant light found up to 23% rhythmic phosphopeptides (Choudhary *et al.*, 2015; Krahmer *et al.*, 2022). These studies suggest that light responses and the circadian clock in Arabidopsis each control 5- to 10-fold more phosphopeptides than the diel rhythm of the total protein level, so it is also important to understand which phospho-regulators mediate these effects.

The amino acid sequences of rhythmically regulated phosphosites have implicated a range of protein kinases with overlapping contributions in Arabidopsis (Choudhary *et al.*, 2015; Uhrig *et al.*, 2021; Krahmer *et al.*, 2022). However, ~1000 protein kinases shape the phosphoproteome in Arabidopsis (Champion *et al.*, 2004) including several in plastids (Baginsky and Gruissem, 2009), compared with half that number in the human genome (Manning *et al.*, 2002). Of particular interest, the casein kinases (CK1 and CK2) and glycogen synthase kinase 3 (GSK3) affect the circadian timing of all organisms studied (Mehra *et al.*, 2009). These kinases have central positions in the yeast kinase–target network (Breitkreutz *et al.*, 2010) and are highly conserved (Hindle *et al.*, 2014), in contrast to photoreceptor proteins or circadian transcription factors (Noordally and Millar, 2015; Dunlap and Loros, 2017).

Here, we compare the prevalence of proteomic and phosphoproteomic regulation under LD cycles, using *O. tauri* as a minimal model for the green lineage (Noordally and Millar, 2015). This alga not only has a ubiquitously rhythmic transcriptome, but its genome is also reduced to 13 Mbp (Blanc-Mathieu *et al.*, 2014), probably due to selection pressure to reduce cell size to 1–2 µm (Courties *et al.*, 1994). Its 7699 protein-coding genes include just 133 protein kinases that represent the core families for eukaryotic signalling (Hindle *et al.*, 2014) and a minimal set of Arabidopsis clock gene homologues (Corellou *et al.*, 2009; Troein *et al.*, 2011; Djouani-Tahri el *et al.*, 2011b; Ocone *et al.*, 2013). CK1 and CK2 modulate circadian timing in the light, with widespread effects on the algal phosphoproteome (Le Bihan *et al.*, 2011, 2015; van Ooijen *et al.*, 2013). A non-transcriptional, 24 h oscillator of unknown mechanism was also revealed under prolonged darkness, when transcription stops in this organism (O’Neill *et al.*, 2011; van Ooijen *et al.*, 2011; Edgar *et al.*, 2012; Bouget *et al.*, 2014; Feeney *et al.*, 2016). In cyanobacteria, the non-transcriptional clock is driven by rhythmic protein phosphorylation, so rhythmic protein kinase activities could also be relevant in *O. tauri* (van Ooijen and Millar, 2012; Wong and O’Neill, 2018).

Our results reveal widespread daily rhythms in both the proteome and phosphoproteome in *O. tauri*, including expected features such as the diel control of conserved, cell cycle phospho-regulators. Rather than the rapid phosphorylation responses and slow, rhythmic anticipation in protein profiles that might be expected, however, much of the rhythmic phosphoproteome anticipates dawn, whereas the level of many rhythmic proteins appears light responsive. The phosphosite sequences strongly implicate phase-specific protein kinase classes. Moreover, we identify a set of rhythmic, algal-specific proteins that accumulate in prolonged darkness and were also identified in conditions that promote the formation of lipid droplets.

## Materials and methods

### Materials

Chemicals were purchased from Sigma-Aldrich (now a subsidiary of Merck Life Science UK Ltd, Dorset, UK) unless otherwise stated. The main solvent, acetonitrile, and water for liquid chromatography–dual MS (LC-MSMS) and sample preparation were HPLC quality (Thermo Fisher Scientific, Loughborough, UK). Formic acid was Suprapure 98–100% (Merck) and trifluoroacetic acid (TFA) was 99% purity sequencing grade. TPCK-treated porcine trypsin was from Worthington (Lorne Laboratories, Reading, UK). All HPLC-MS connectors and fittings were from Upchurch Scientific (Hichrom, Theale, UK) or Valco (RESTEK, High Wycombe, UK). Percentages are expressed in v/v.

### O. tauri media and culturing

*Ostreococcus tauri* OTTH95 were cultured as previously described (van Ooijen *et al.*, 2012), supplemented with 0.22 μm-filtered 50 µg ml^–1^ ampicillin, neomycin, and kanamycin antibiotics in vented tissue culture flasks (Sarstedt, Leicester, UK). Cultures were maintained by splitting weekly at a 1:50 dilution. In preparation for proteomics experiments, cultures were grown in growth medium supplemented with 200 mM sorbitol and 0.4% glycerol for 7 d prior to the start of harvesting (O’Neill *et al.*, 2011). Cells were cultured under cycles of 12 h light/12 h dark at 20 °C in a controlled-environment chamber (MLR-350, Sanyo Gallenkamp, Loughborough, UK) at a light intensity of 17.5 μmol m^–2^ s^−1^ white fluorescent light filtered by a 724 Ocean Blue filter (LEE Filters Worldwide, Andover, UK).

### O. tauri cell harvesting

Cells were grown for 7 d in LD conditions and, on the seventh day, five replicate cultures were harvested per time point, at Zeitgeber times (ZTs) 0, 4, 8, 12, 16, and 20, where ZT0 corresponds to dawn. At ZT0, cells were harvested a few minutes before the lights went on and, at ZT12, before the lights went off. A 135 ml culture was harvested by centrifugation (4000 rpm, 10 min, 4 °C) per sample replicate, each from a separate culture vessel. Pellets were resuspended in ice-cold phosphate-buffered saline (PBS). Cultures were centrifuged as before; pellets were air dried, vortex mixed in 250 µl of 8 M urea, and stored at –80 °C. For total cell lysate, cells were dissolved by sonication (Branson Ultrasonics) and diluted with 500 µl of dH_2_O.

Cells were grown for 7 d in LD conditions and, on the eighth day the dark adaptation (DA) experiment cell harvests were performed at ZT24, 48, 72, and 96 in constant darkness with five replications. The samples were harvested and prepared as for the LD experiment.

### Protein digestion

Samples were analysed by Bradford Assay (Bio-Rad, Watford, UK) and 400 µg of protein of each sample was used in the digestion. Samples were reduced in 10 mM DTT and 50 mM ammonium bicarbonate, and alkylated with 25 mM iodoacetamide. Samples were digested overnight with 10 µg (1:40 ratio) of trypsin under agitation at room temperature at pH 8 in a total volume of 1 ml. Samples were cleaned on solid phase extraction (SPE) BondElut 25 mg columns (Agilent Technologies, Stockport, UK) following the vendor’s instruction. A 50 µl aliquot (~20 µg) was removed and dried for LC-MS (Speedvac, Thermo Fisher Scientific). The remaining ~380 µg were also dried in preparation for phosphopeptide enrichment, and stored at –20 °C.

### Phosphopeptide enrichment

Dried peptide samples (~380 µg) were sonicated in 50 µl of solution 0 (2.5% acetonitrile, 0.5% TFA) and 100 µl of solution 2 (80% acetonitrile, 0.5% TFA, 100% lactic acid). Titansphere Phos-TiO Kit spin tip-columns (GL Sciences, Tokyo, Japan) were washed with 40 µl of solution 1 (80% acetonitrile, 0.5% TFA). Samples were loaded on the spin tip-columns and passaged three times through a centrifuge; 5 min at 200 *g*, 15 min incubation at room temperature, and 10 min at 200 *g*. Spin tip-columns were subsequently washed once with solution 1, twice with solution 2, and twice with solution 1 for 2 min at 200 *g*. Phosphopeptides were eluted in two steps, first with 50 µl of 5% ammonium hydroxide (5 min at 200 *g*) and secondly with 5% pyrrolidine solution. A 20 µl aliquot of 20% formic acid was added to lower the pH, and samples were cleaned on Bond Elut OMIX C18 pipette tips (Agilent Technologies) following the manufacturer’s instruction.

### Protein and phosphoprotein quantification

A 15 µg aliquot of protein from total *O. tauri* cell lysates was run on a Novex NuPAGE 4–12% Bis-Tris by SDS–PAGE with PeppermintStick Phosphoprotein Molecular Weight Standards and a Spectra Multicolor Broad Range Protein Ladder (Thermo Fisher Scientific). The gel was fixed overnight (50% methanol, 40% ddH_2_O, 10% glacial acetic acid), washed in ddH_2_O, and stained with Pro-Q Diamond Phosphoprotein Gel Stain (Invitrogen, now Thermo Fisher Scientific, Loughborough, UK) in the dark at 25 °C following the manufacturer’s instructions. The gel was imaged on a Typhoon TRIO variable mode imager (GE Healthcare, Amersham, UK) at 532 nm excitation/580 nm emission, 450 PMT, and 50 µm resolution. Images were processed using ImageQuant TL software (GE Healthcare, Amersham, UK). The gel was re-used for protein quantification using SYPRO Ruby Protein Gel Stain (Thermo Fisher Scientific) following the manufacturer’s instructions and imaged using a UV transilluminator (Ultra-Violet Products Ltd, Cambridge UK). Protein and phosphoprotein bands were quantified using Image Studio Lite v 4.0 (LI-COR Biosciences, Cambridge, UK).

### Protein per cell quantification

Cells were grown (as described above) and independent, triplicate cultures were harvested at the times indicated. Cultures were monitored using spectrophotometry at 600 nm. Total protein was quantified using the Quick Start Bradford Assay following the manufacturer’s instructions (Bio-Rad, Watford, UK). Cell number was estimated either by counting four fields of view per culture in a haemocytometer after trypan blue staining (Abcam protocols, Cambridge, UK), or by fluorescence-activated cell sorting (FACS). For FACS, a 1/200 dilution of cells was transferred to fresh medium containing 1× SYBR Green I Nucleic Acid Gel Stain (Invitrogen, now Thermo Fisher Scientific) and FACS counted (FACScan, BD Bioscience, Wokingham, UK) at a flow rate of 60 μl min^–1^.

### Quantitative PCR for transcriptional regulation during dark adaptation

Cells were cultured and harvested using the same experimental regime (described above) and harvested in biological triplicate at the times indicated for the LD and DA experiments. Total RNA was extracted from frozen cells using an RNeasy Plant Mini Kit and DNase treated (QIAGEN, Manchester, UK). First-strand cDNA was synthesized using 1 µg of RNA and 500 ng µl^–1^ oligo(dT)_15_ primer (Promega, Southampton, UK), denatured at 65 °C for 5 min, and reverse transcribed using SuperScript II (Invitrogen, now Thermo Fisher Scientific) at 42 °C for 50 min and 70 °C for 10 min. cDNA dilutions (1/100) were analysed using a LightCycler^®^480 and LightCycler^®^480 SYBR Green I Master (Roche, Welwyn Garden City, UK) following the manufacturer’s instructions and cycling conditions of pre-incubation at 95 °C for 5 min; 45× amplification cycles of 95 °C for 10 s, 60 °C for 10 s, 72 °C for 10 s. The following 5ʹ to 3ʹ forward (F) and reverse (R) primers to *O. tauri* gene loci were used: ostta01g01560, GTTGCCATCAACGGTTTCGG (F), GATTGGTTCACGCACACGAC (R); ostta03g00220, AAGGCTGGTTTGGCACAGAT (F), GCGCTTGCTCGACGTTAAC (R); ostta03g04500, GCCGCGGAAGATTCTTTCAAG (F), TCATCCGCCGTGATGTTGTG (R); ostta04g02740, ATCACCTGAACGATCGTGCG (F), CCGACTTACCCTCCTTAAGCG (R); ostta10g02780, GGCGTTCTTGGAATCTCTCGT (F), TATCGTCGATGATCCCGCCC (R); ostta10g03200, GGTACGGAGGAAGAAGTGGC (F), ATGTCCATGAGCTTCGGCAA (R); ostta14g00065, GACAGCCGGTGGATCAGAAG (F), TCGAGGTAGCTCGGGAGATC (R); ostta16g01620, ACGGGTTGCAGCTCATCTAC (F), CCGCTTGGGTCCAGTACTTC (R); ostta18g01250, CTTGCAAATGTCCACGACGG (F), ATGATGTGGCACGTCTCACC (R); OtCpg00010, ACATGACTCACGCGCCTTTA (F), TGCCAAAGGTGCCCTACAAA (R). Primers to eukaryotic translation elongation/initiation factor (EF1a) ostta04g05410 GACGCGACGGTGGATCAA (F) and CGACTGCCATCGTTTTACC (R) were used as an endogenous control. Primers to eukaryotic translation elongation/initiation factor (EF1a) were used as an endogenous control. This transcript is among the least varying 1% of the transcriptome tested by RNAseq under LD cycle conditions (Derelle *et al.*, 2018). Data were combined for biological and two technical replicates, and relative quantification was performed using LightCycler^®^480 1.5 software (Roche).

### HPLC-MS analysis

Micro-HPLC-MS/MS analyses were performed using an on-line system consisting of a micro-pump 1200 binary HPLC system (Agilent Technologies) coupled to a hybrid LTQ-Orbitrap XL instrument (Thermo Fisher Scientific). The complete method has been described previously (Le Bihan *et al.*, 2010). For all measurements, 8 µl of sample was injected using a micro-WPS auto sampler (Agilent Technologies) at 5 µl min^–1^. After sample loading, the flow rate across the column was reduced to ~100–200 nl min^–1^ using a vented column arrangement. Samples were analysed on a 140 min gradient for data-dependent analysis.

### HPLC-MS data analysis

To generate files compatible with the public access databases PRIDE (Vizcaino *et al.*, 2016) and the former pep2pro (Hirsch-Hoffmann *et al.*, 2012), Mascot Generic Format (MGF) input files were generated using MSConvert from ProteoWizard (Kessner *et al.*, 2008). MSMS data were searched using MASCOT version 2.4 (Matrix Science Ltd, London, UK) against the *O. tauri* subset of the NCBI protein database (10 114 sequences from NCBI version 6 June 2014 including common contaminants) using a maximum missed­cut value of 2, variable oxidation (M), N-terminal protein acetylation, phosphorylation (STY), and fixed carbamidomethylation (C); precursor mass tolerance was 7 ppm and MSMS tolerance 0.4 amu. The significance threshold (*P*) was set below 0.05 (MudPIT scoring). The global false discovery rate (FDR) was evaluated using a decoy database search and removal of peptides ranked higher than 1 for a mascot score >20 (~1% global FDR). MS proteomics data have been deposited in the PRIDE ProteomeXchange Consortium (Vizcaino *et al.*, 2014) via the PRIDE partner repository with the dataset identifier LD global proteomics, PXD001735; LD phosphoproteomics, PXD001734; DA global proteomics, PXD002909. Data were converted into PRIDEXML using Pride converter 2.0.20 and submitted using the proteome exchange tool pxsubmission tool 2.0.1. The LC-MS data are also publicly available in the former pep2pro database (Assemblies ‘Ostreococcus tauri Light:dark cycle,LD global’, ‘Ostreococcus tauri Light:dark cycle,LD phospho’, and ‘Ostreococcus tauri dark adaptation,DA global’).

Label-free quantification was performed using Progenesis version 4.1 (Nonlinear Dynamics, Newcastle, UK). Only MS peaks with a charge of 2+, 3+, or 4+ and the five most intense spectra within each feature were included in the analysis. Peptide abundances were mean-normalized and arcsinh-transformed to generate normal datasets. Within-group means were calculated to determine fold changes. Neutral losses of phosphoric acid typical of serine and threonine being phosphorylated were validated manually in all significantly differential phosphopeptides. Ambiguous sites were confirmed by cross-referencing (by sequence, charge, and quantity of residue modifications) with most probable site predictions from MaxQuant version 1.0.13.8 (Cox and Mann, 2008) in singlet mode, with Mascot settings as above. Where multiple occurrences of residue phosphorylation events were quantified, abundances were summed, collating all charge states, missed cuts, and further modifications.

### Data analysis

#### Merging

For accurate and unique phosphopeptide quantification, we addressed variant redundancy at different charge states, alternative modifications (e.g. oxidation and acetylation), and multiple sites of protease digestion. All unique phosphorylation events were retained, including multiple phosphorylation, at a given amino acid motif, while summing the quantification of these technical variants. The qpMerge (http://sourceforge.net/projects/ppmerge/) software was used to combine Progenesis and MaxQuant phospho-site predictions and produce a unique set of quantified phosphopeptide motifs (Hindle *et al.*, 2016, Preprint).

#### Outlier identification and removal

To detect outliers, we first applied principal component analysis (PCA) to all the replicates and then calculated the Pearson correlation of each replicate’s data to the median abundance values from all five replicates at that time point. A single phosphoproteomic replicate, 4E, was excluded based on substantial differences in peptide quantification that led to *r*^2^<0.8 (Supplementary Fig. S1D).

#### P-value calculation and false discovery rate

For analysing the significance of changing protein and peptide abundance over time, non-linear response of expression using polynomial regression was modelled using the R Stats Package. A third-order polynomial was fitted, testing for an expected peak and trough within a 24 h daily cycle against the variation among replicates. This approach avoided the manual removal of continually rising or falling traces, which was previously required when JTKcycle was used to score rhythmicity within a single cycle of data (Krahmer *et al.*, 2022). An arcsinh transformation of abundance was applied to meet the required assumption of normality (Burbidge *et al.*, 1988). FDR was calculated using the Benjamini and Hochberg (BH) method (Benjamini and Hochberg, 1995). More than two quantifying peptides were required to report protein abundance.

#### Equivalence testing

Using the R equivalence package, the statistical equivalence of mean abundance across time was tested as the highest *P*-value from exhaustive pairwise two one-sided test approach (TOST) tests over all ZTs (Schuirmann, 1981; Westlake, 1981). We tested whether abundances had upper and lower differences of <0.3 within the equivalence margin (ε).

#### O. tauri gene identifiers

The *O. tauri* genome version 1 gene IDs (Derelle *et al.*, 2006) for microarray data were converted to version 2 IDs (Blanc-Mathieu *et al.*, 2014) by finding exact sequence matches for the microarray probes (accession GPL8644) (Monnier *et al.*, 2010) in the version 2 FASTA coding sequence file.

#### Principal component analysis

PCA was used to investigate the main components of variation in the data using prcomp from the R Stats Package. The abundances were zero-centred per feature. The PCA loading values for each feature were extracted and then used for Gene Ontology (GO) enrichment analysis.

#### Clustering

Hierarchical clustering was performed with hclust from the R Stats Package and applied on all per-feature (protein or phosphopeptide motif) mean abundances over time, which were zero-centred and scaled. Pearson’s correlation was used to calculate the distance matrix and the Ward method (Ward, 1963) for linkage criteria. The hierarchical tree was divided into clusters using the dynamicTreeCut algorithm (Langfelder *et al.*, 2008). The hybrid cut tree method with a cut height of 100 and a minimum cluster size of 20 was used for both datasets.

#### Enrichment analysis for GO terms

TopGO was used to evaluate the enrichment of GO terms, for each ontology aspect, within clusters, peaks, troughs, and principal components (PCs). The peak (or trough) time is the time point with the maximum (minimum) mean level in the experiment. For clusters, peaks, and troughs, a Fisher’s exact test was used by partitioning at 95% confidence on FDR-corrected *P*-values, and with a fold change >1.5 in normalized abundance. For each test, we use a relevant background of non-significant observed features. For PCs, variable loadings quantify how much each protein/phosphopeptide motif contributes to (or weights) the variance captured by the PC. GO enrichment using these variable loadings tests for terms that are statistically over-represented among the proteins/phosphopeptide motifs with higher loading in the PC. To test for enrichment of GO terms for each PCA, the Kolmogorov–Smirnov test was applied over the absolute PCA loading values for each gene. GO terms were predicted by InterProScan 5 (Jones *et al.*, 2014) on amino acid sequences for *O. tauri* coding sequences [NCBI version 140606 (Blanc-Mathieu *et al.*, 2014)].

#### Homology modelling

Structural homology models were generated using I-TASSER (Yang and Zhang, 2015) for prasinophyte family-specific proteins of unknown structure and function, including for ostta02g03680 compared with the human Bar-domain protein structure in the PDB entry with DOI 10.2210/pdb2d4c/pdb. Other suggested homologies were more limited.

#### pLOGO and binomial statistics

Significantly over- and under-represented amino acid residues at different time points were calculated using the binomial-based pLogo tool (O’Shea *et al.*, 2013). The Motif-X tool (Chou and Schwartz, 2011) was used to discover novel motifs in the dataset. Binomial statistics were applied to calculate the enrichment of motifs and the combined probabilities of amino acids with similar properties in a phosphopeptide motif (e.g. the acidic D/E positions in the CK2 motif).

#### Kinase target prediction

Computational prediction of protein kinase motifs associated with the identified phosphorylation sites was performed using Group-based Prediction System, GPS Version 3.0 (http://igps.biocuckoo.org/index.php) (Xue *et al.*, 2011).

#### O. tauri loci IDs mapping to A. thaliana loci IDs

The *O. tauri* and *A. thaliana* IDs were mapped using EggNOG4.1 (http://eggnogdb.embl.de). The *O. tauri* proteins were downloaded from https://bioinformatics.psb.ugent.be/gdb/ostreococcusV2/LATEST/OsttaV2_PROT_20140522.fasta.gz (22 May 2014). Viridiplantae (virNOG) hmms and their descriptions and annotations were transferred to *O. tauri* proteins using hmmr 3.1 (http://hmmer.janelia.org).

### Mathematical simulations

#### Simulated protein rhythms

Protein dynamics [*P*(*t*)] were simulated according to the following model:


$$\frac{\text{d}P\left(t\right)}{\text{d}t}=\left(\left(k_{\text{syn}}-1\right)L+1\right)m\left(t\right)-k_{\text{deg}}P\left(t\right)$$

Where *L*(*t*)=1 during the day (ZT ≤12), and 0 otherwise. The rate of protein degradation (*k*_deg_) was set to 0.1 h^–1^, and the ratio of protein synthesis in the light compared with the dark (*k*_syn_) was set to 4, based on Martin *et al.* (2012), for all the simulated proteins. We note that protein turnover could also be modelled to include a varying rate of dilution. However, this effect is small relative to the degradation rate modelled here (average dilution across a 24 h period of 0.01 h^–1^, with variation of this rate across the period being less than this). The rhythmically expressed mRNA levels [*m*(*t*)] are given by:


$$m(t\text{)}=\cos\left(\frac{2\text{​​π​​ }\left(t-\text{ ​​}\varphi \text{​​ }\right)}{24}\right)+1$$

The peak phase of expression is given by φ. To obtain the distributions of peak and trough protein levels, the peak phases (φ) of mRNA expression were uniformly distributed at 0.1 h intervals across the range [0,24]. For each phase of mRNA expression, the timing of peak and trough protein levels was determined by simulating the model dynamics in MATLAB using the ode15s ODE solver. The peaks and troughs were identified across a 24 h period, following 240 h simulation to allow the dynamics to reach a steady behaviour (i.e. with the same protein levels at ZT0 and ZT24).

#### Protein degradation rates and depletion during dark adaptation

Degradation rates were calculated from published proteomics data (Martin *et al.*, 2012), which characterized the dynamics of partial ^15^N isotope incorporation. We assumed a labelling efficiency of 0.93 (=maximum labelled fraction achieved of any protein +0.01), and fitted a simple kinetic model assuming: (i) constant labelling efficiency over time; (ii) different proteins are labelled at the same efficiency; and (iii) heavy and light fractions are turned over at equal rates, similar to Seaton *et al.* (2018). In calculating the correlation of the resulting degradation rates with fold change under dark adaptation (Fig. 3C), we considered potential outliers. One protein (ostta02g04360) with a high degradation rate (~0.03 h^–1^) and fold change (~0.5) was excluded as an outlier, as including this single protein significantly increased the degree of anticorrelation (Pearson’s correlation coefficient changed from *r*= –0.48 to –0.7 when included). The two proteins with the next highest fold changes (~0.6, ostta10g03200 and ostta14g02420) were retained; excluding these proteins also would change the correlation to *r*= –0.39, which would remain significant (*P*=0.02).

## Results

**Fig. 1.**
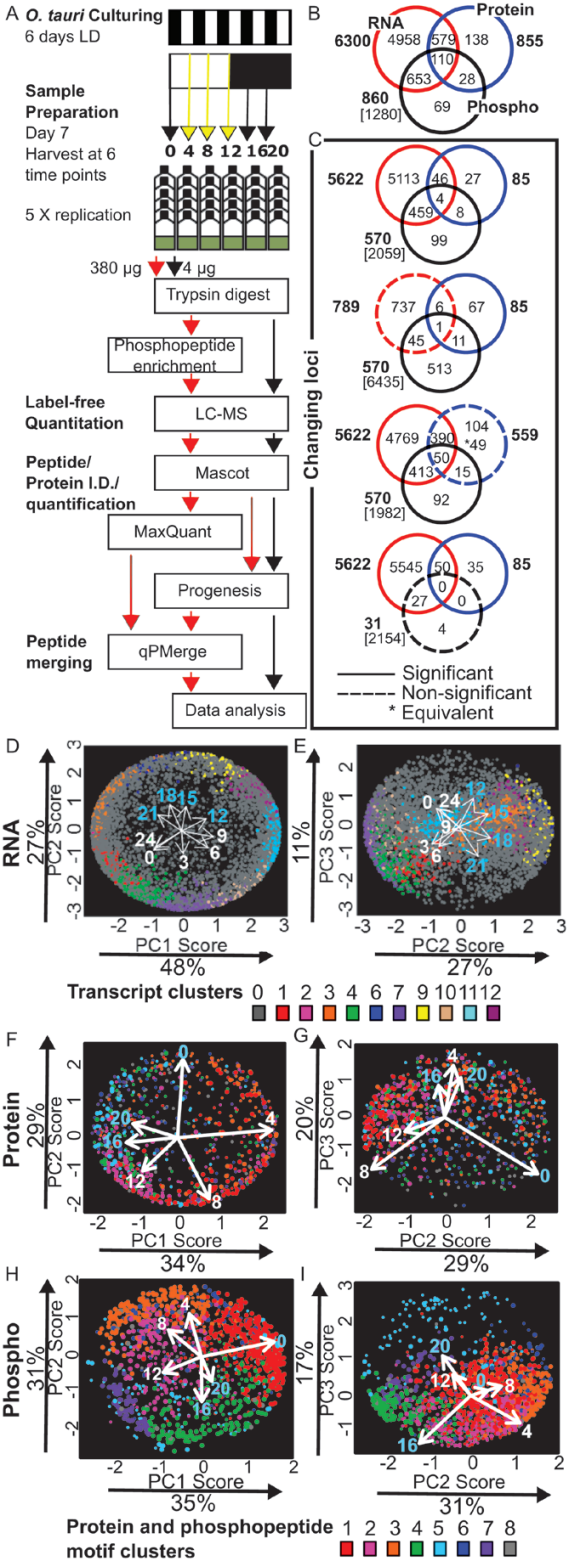
Daily variation in transcripts, proteins, and phosphopeptide motifs. (A) Workflow for proteomics in *O. tauri* under LD cycles. Overlap in (B) detected and quantified gene loci. (C) Significantly changing (filled circles) or not significantly changing (dashed circles) loci for transcripts (Monnier *et al.*, 2010), proteins, and phosphopeptide motifs; genomic loci were excluded (square brackets). (D–I) Bi-plots of PCA for the time series of mean levels of each (D, E) transcript, (F, G) protein, and (H, I) phosphopeptide motif. The proportion of the variance for each PC is indicated. Dot locations show the weighting of each RNA/protein/phosphopeptide motif time series in each PC; colours show the assigned cluster (as in Supplementary Fig. S4A, B). Loading arrows show the magnitude (by length) and relative contribution (by direction) of data from each time point to the PCs that are plotted, hence the angles between loading arrows indicate correlation (0°) and anticorrelation (180°).

To understand the landscape of protein abundance and phosphorylation across the diel cycle, we harvested quintuplicate biological samples of *O. tauri* at six time points across a 12 h light/12 h dark (LD) cycle. Dawn samples (ZT0) were harvested just before lights on, and samples at ZT12 before lights off, to detect biological regulation that anticipated these transitions. The proteome and phosphoproteome were measured in whole-cell extracts from each sample, by label-free, LC-MS (Fig. 1A). A total of 855 proteins were quantified with two or more peptides (Supplementary Table S1). Phosphopeptides were enriched by metal affinity chromatography prior to detection. For quantification, we combined the phosphopeptide species that shared phosphorylation on a particular amino acid, irrespective of other modifications (Hindle *et al.*, 2016, Preprint). We refer to this set of phosphorylated species as a phosphopeptide motif. After removing a technical outlier (Supplementary Fig. S1), 1472 P phosphopeptide motifs were quantified, from 860 proteins (Supplementary Table S2). Serine and threonine residues were modified most; only 1% of phosphopeptide motifs included phospho-tyrosine. The quantified proteins and phosphoproteins each represent ~11% of the total *O. tauri* proteome (Fig. 1B). A total of 29 out of 61 proteins encoded on the chloroplast genome (Robbens *et al.*, 2007) were quantified, with six phosphopeptide motifs. Three out of 43 mitochondrial-encoded proteins were quantified with no phosphopeptide motifs, consistent with other studies (Ito *et al.*, 2009).

### Diel rhythmicity of the transcriptome, proteome, and phosphoproteome

To compare the patterns and prevalence of daily rhythms at different regulatory levels, we re-analysed published transcriptome data in parallel with these protein and phosphoprotein data, summarized in Fig. 1C. Gene expression in *O. tauri* was strongly rhythmic under LD cycles, with 89% of transcripts scored as rhythmic, as previously reported (Monnier *et al.*, 2010). Eighty-five (9.5%) of the detected proteins were significantly rhythmic by polynomial regression (see the Materials and methods) and changed by at least 1.5-fold, with only 11 of these proteins changing level by >5-fold. In contrast, 66% of phosphoproteins or 58% of phosphopeptide motifs (570 of 860 proteins; 850 of 1472 phosphopeptide motifs) were rhythmic by these criteria and the levels of 35 phosphopeptide motifs changed >20-fold. These results show more rhythmicity in the levels of detected RNAs and phosphopeptide motifs than in protein levels. Understanding how a specific gene of interest was regulated, however, was hampered by the fact that only 110 genes were quantified in all three datasets (Fig. 1C).

Protein levels nonetheless changed smoothly, with distinct waveforms. Of the 20 most highly detected proteins, probably including the most abundant, 11 were significantly rhythmic but with low amplitudes (Supplementary Fig. S2A), such that only ostta10g03200 exceeded the 1.5-fold change threshold (Supplementary Table S1). Fifteen of the 20 most highly detected phosphopeptide motifs, in contrast, were rhythmic by both criteria (Supplementary Fig. S2B). The more stringent, ‘equivalence’ test revealed 49 proteins with significantly non-changing protein abundance but with significantly changing transcript and phosphopeptide motifs, illustrated by the 10-fold change in phosphopeptide motif abundance on the non-changing chlorophyll-binding protein CP26, amongst others (Supplementary Fig. S3).

### Contrasting patterns of regulation

To identify the dominant patterns of regulation (anticipatory, reactive, or otherwise), we applied undirected PCA to the mean level of each RNA, protein, or phosphopeptide motif at each time point (Fig. 1D–I). The PCA represented most (83–86%) of the variance in each dataset but indicated a differing balance of molecular regulation between them. The transcriptome and phosphoproteome data clearly separated between dawn and dusk time points in PC1, and between the light and dark intervals in PC2. That separation also mapped the contributions of the 13 transcriptome and 6 phosphoproteome time points, each indicated by an arrow on the figure panels, into their respective, temporal sequences, as expected if smoothly changing time series are prominent in the data. The lesser contributions from PC3 separated some adjacent time points such as ZT0/24 from ZT3 in the RNA, and ZT16 from ZT20 in the phosphopeptide motifs, indicating contrasting profiles between these time points, but PC3 results were otherwise harder to interpret. The PCA results for RNA and phosphopeptide motif molecular profiles suggested anticipatory rather than responsive regulation, because the strongest effects (in PC1) corresponded to time of day, not the light/dark condition of each sample.

The relatively few rhythmic proteins, in contrast, showed evidence of reactive, not anticipatory, regulation. The major separation (in PC1) was between samples from light and dark intervals (Fig. 1F, G). The early day (ZT4) was separated most strongly from early night to mid night (ZT16 and 20). The lower contribution of PC2 separated the late night (ZT0) from the late day (ZT8–12). PC3 was not easily interpretable, though it accounted for 20% of the variance, probably reflecting the low amplitude of the protein regulation observed.

**Fig. 2.**
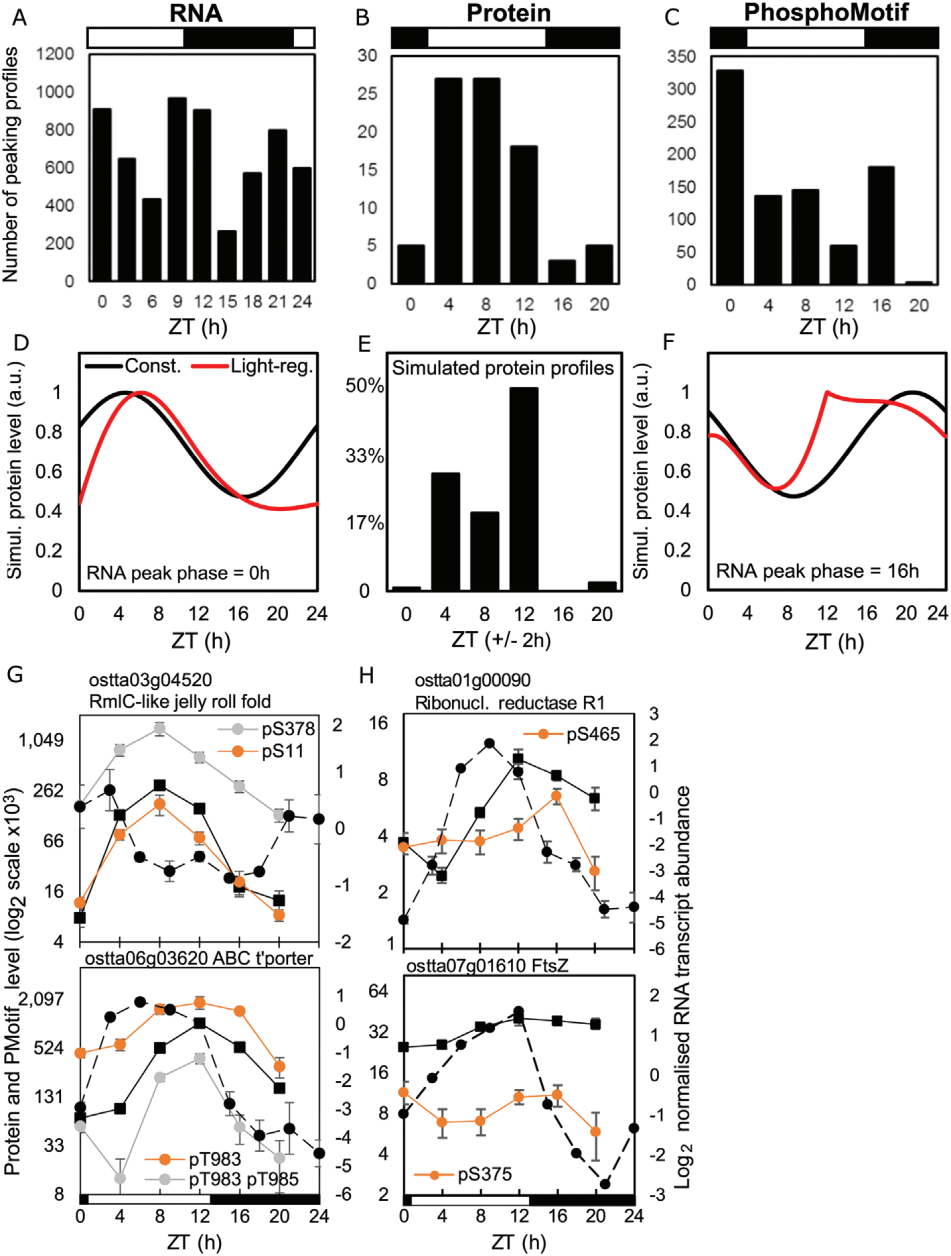
Distribution of rhythmic protein and phosphopeptide motif peaks, with examples. Temporal distribution of peaking profiles in (A) transcripts, (B) proteins, and (C) phosphopeptide motifs. (D, F) Simulated protein profiles from RNAs peaking at (D) ZT0 or (F) ZT16, with (red line) or without light-regulated translation (black line). (E) Predicted distribution of protein peak times, with light-regulated translation. Examples of genes with (G) high-amplitude and similar protein (solid line) and phosphopeptide motif profiles (coloured lines), or (H) phosphopeptide motif profiles that differ from the protein profile. (G, H) Protein and phosphopeptide motif, left axis; RNA profile (dashed line), right axis. Error bars, SE. Light/dark are indicated by white/black bars.

Clustering (Fig. 1D–I; Supplementary Fig. S4) and analysis of peak distributions (Fig. 2A–C) informed more detailed hypotheses on upstream regulation and downstream functional effects. Hierarchical clustering grouped the protein and phosphopeptide motif abundance profiles into eight clusters (termed P1–P8 and PM1–PM8, respectively; Supplementary Fig. S4A, B). The consistency among the analysis methods is illustrated in Fig. 1D–I. The coordinates of RNAs or phosphopeptide motifs in the PCA plots align with their separation into distinct clusters, represented by the colour of each RNA or phosphopeptide motif marker, and with particular time points. For example, the phosphopeptide motif profiles with large positive values in PC1 (Fig. 1H) also correspond to the contributions of the pre-dawn time point ZT0 (indicated by the arrow, Fig. 1H) and to membership of cluster PM1 (red markers, as in Supplementary Fig. S4B). Clustering of the lower amplitude, protein profiles did not align so clearly with the PCA (Fig. 1F, G).

GO term enrichment data for RNAs, proteins, and phosphopeptide motifs in the PC clustering and peak time analyses is presented in Supplementary Tables S3–S5, with examples for proteins and phosphopeptide motifs in Supplementary Fig. S4C, D and a summary in Supplementary Fig. S5. Results for RNAs were similar to past analysis of these data (Monnier *et al.*, 2010), as expected. The section ‘Functions of proteins with rhythmic phosphomotifs’ outlines the functional analysis of phosphopeptide motifs. Among the rhythmic protein functions, proteins involved in the TCA cycle and transport processes were enriched in PC2, aligned with the late night (ZT0). PC1 was notably enriched for translation-related protein functions, which had previously been highlighted in transcript profiles peaking after dawn (Monnier *et al.*, 2010). Our next analysis suggested the functional effect of translational regulation.

### Daytime peaks of protein abundance

We analysed the distribution of peak times among the rhythmic profiles (Fig. 2) to understand the anticipatory or reactive regulation in more detail, starting with the proteins. Hundreds of transcripts reach peak abundance at every time point around the day/night cycle (Fig. 2A) (Monnier *et al.*, 2010). In contrast, most protein profiles peaked in the light interval (85% at ZT4–12; Fig. 2B), separating the day and night samples in line with the PCA. Metabolic labelling of *O. tauri* has shown ~5-fold higher protein synthesis rates in the day compared with the night (Martin *et al.*, 2012). Consistent with this, our analyses showed that translation-related proteins were enriched among the rhythmic proteins with high abundance in the daytime, whether in PC1, protein cluster P1, or in profiles with daytime peak phase (Supplementary Tables S3–S5; Supplementary Figs S4, S5). We therefore tested whether this light-regulated synthesis alone could explain the observed distribution of protein peak times.

We simulated protein dynamics (Fig. 2D–F; Supplementary Fig. S6) using measured protein synthesis and degradation rates (Martin *et al.*, 2012), and an even temporal distribution of peak times among a population of simulated, rhythmic mRNAs. Without light regulation, the translation of these rhythmic RNAs would result in a corresponding even distribution of peak times across the day and night in the protein profiles also (black traces, Supplementary Fig. S6A–C), with each protein profile following its cognate RNA. With the observed light regulation, however, the simulated distribution of protein profiles matched well to the high proportion of daytime peaks in our measured protein profiles (Fig. 2E; Supplementary Fig. S6D–G). The concentration of simulated protein peaks in daytime time points (97%, compared with 85% in the data) emphasizes the strength of the translational effect. ostta03g04520 is an example of an RNA that peaks at ZT0, and its protein profile (Fig. 2G) was very similar to the predicted protein from such an RNA (Fig. 2D). Proteins in cluster P4 (Supplementary Fig. S4A) might also reflect light-stimulated translation as they reach peak levels at ZT12, similar to the simulated example in Fig. 2F. The overall distribution of protein profiles substantially reflects the light-stimulated translation rate of this organism (see the Discussion).

### Unusual, night-time proteins suggest a ‘dark state’

**Fig. 3.**
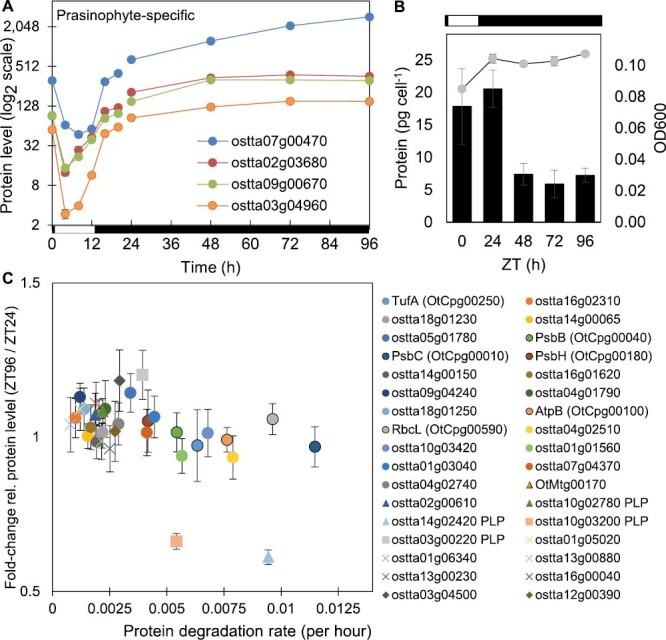
Regulation of dark-accumulating proteins. Protein abundance profiles (A) of rhythmic prasinophyte-specific proteins in cluster P6 in LD and DA conditions. (B) Optical density (OD_600_; line, right axis) and total protein per cell (columns, left axis) under LD and DA conditions. (C) Correlation of protein degradation rates (Martin *et al.*, 2012) and relative protein levels after DA; chloroplast proteins (circles, chloroplast-encoded a have solid outline); mitochondrial proteins (triangles, mitochondria-encoded outlined); PLP-enzymes (squares, marked in key); prasinophyte-specific proteins (diamonds).

An intriguing pattern of protein regulation stood out from the daytime abundance of rhythmic proteins. Protein cluster P6 included the protein profiles that fell at ZT4 (Supplementary Fig. S4A), associated with oxidative metabolism and protein transport GO terms (Supplementary Table S4). Four unannotated proteins in cluster P6, with sequence homologues only among the prasinophyte group of green algae, not only peaked at night but were also among the 11 highest amplitude profiles of all the rhythmic proteins (Fig. 3A). Their dramatic fall in abundance at ZT4 suggested a destabilization by light, so we tested whether such proteins would remain stable during several days of DA.

*Ostreococcus tauri* cells are photo-autotrophic. Their division is entrained by the LD cycle (Farinas *et al.*, 2006) and they arrest transcription in prolonged darkness, when they can survive without growth or division if sorbitol and glycerol are provided in the medium (O’Neill *et al.*, 2011). Cell density (optical density at 600 nm) in our cultures increased by ~25% after one LD cycle. Cellular protein content was consistent (18–20 pg per cell) in replicate measures at ZT0 and ZT24 (Fig. 3B). In cultures transferred to a further 3 d of darkness, the optical density remained constant but protein content per cell dropped by >60% on the first day (ZT24–ZT48) and was then stable to ZT96. This result was suggestive of an altered, but potentially stable, cellular ‘dark state’, which we tested in a further, proteomic time series, sampling in darkness at ZT24, 48, 72, and 96.

The proteomic landscape changed less during DA than under a standard LD cycle. A total of 98 of the 865 proteins quantified by LC-MS changed levels more than the average and only 64 (7%) also changed >1.5-fold (Supplementary Table S6). The 35 significantly increasing proteins in DA included five transmembrane transporters, a Lon-related protease, and two superoxide dismutases, suggestive of nutrient acquisition, protein mobilization, and oxidative stress responses. The four prasinophyte-specific proteins noted above were among the 10 most increasing proteins in DA, confirming their unusual regulation and suggesting a shared function both at night-time in our LD conditions and in the putative ‘dark state’. The most decreasing among 63 significantly decreasing proteins in DA was a starch synthase (ostta06g02940). Its abundance declined in the night under LD cycles, as did all 10 of the DA-decreasing proteins that were also rhythmic in LD cycles. The largest functional group of depleted proteins comprised 22 cytosolic ribosomal proteins and translation factors (Supplementary Table S6), suggesting that *O. tauri* selectively mobilized this protein pool in darkness.

The night-abundant, prasinophyte proteins that accumulated in DA, and night-depleted proteins that fell in DA (such as ostta06g02940, noted above, Supplementary Table S6; or PPDK ostta02g04360, Supplementary Fig. S7C) suggested that prolonged darkness preserved a night-like state. An alternative explanation was that protein stability in general was altered in the putative dark state. We sought to test that notion, using the protein degradation rates that were previously measured by metabolic labelling in LD conditions (Martin *et al.*, 2012). Falling protein abundance under DA was significantly correlated with higher degradation rates in LD conditions (Fig. 3C; *r*= –0.48, *P*=0.004, *n*=34), even among these abundant, stable proteins. We also tested RNA abundance for a subset of these proteins in DA by qRT-PCR, showing stable levels after 1 d of prolonged darkness (ZT48; Supplementary Fig. S8A). The lack of RNA regulation seemed consistent with the lack of transcription in these conditions (O’Neill *et al.*, 2011). For example, a further prasinophyte-specific protein ostta03g4500 with a stable RNA level and slightly increasing protein level in DA also had a low protein degradation rate in LD cycles (Fig. 3C), and was among the most detected proteins in these conditions (Supplementary Figs S2A, S8B). The RNA data and protein degradation rates suggested that the prasinophyte-specific proteins accumulated due to a focused, regulatory mechanism, rather than generalized refactoring of the proteome.

A pre-print (Smallwood *et al*., 2018a, Preprint) coincident with our first report (Noordally *et al.*, 2018, Preprint) showed that three of the night-expressed, prasinophyte-specific proteins accumulated strongly in *O. tauri* under LD cycles when the growth medium was depleted of nitrogen, particularly if carbon availability was also increased (ostta03g04500 accumulated most; ostta09g00670, third; ostta02g03680, fifth). The third most depleted protein in their conditions was the same starch synthase (ostta06g02940) that fell most in abundance under our prolonged dark treatment. Smallwood *et al.* also showed that *O. tauri* forms both intracellular and extracellular lipid droplets under their conditions (Smallwood *et al.*, 2018a, Preprint, 2018b, Preprint). It is possible that sorbitol and glycerol from our medium were metabolized to lipids, and that the night-expressed proteins contributed to that process (see the Discussion).

### A phospho-dawn of protein modification

In contrast to the many daytime-peaking protein profiles, 39% of the changing phosphopeptide motifs peaked in abundance at ZT0 (Fig. 2C), double the proportion of any other time point. The ZT0 samples were harvested before lights on, so this ‘phospho-dawn’ anticipated the dark–light transition and did not reflect increasing protein levels due to light-stimulated translation. In contrast, Fig. 2G shows examples of high-amplitude phosphopeptide motif profiles that did track the levels of their cognate proteins, with little evidence of regulated phosphorylation. We therefore tested the contribution of protein levels to phosphopeptide motif profiles more broadly, among the 138 genes that were quantified in both protein and phosphopeptide motif datasets (Supplementary Fig. S7A, B). This subset of 261 protein-phosphopeptide motif pairings included proteins peaking at all time points, and phosphopeptide motif profiles that reflected the peak time distribution of the full dataset. A total of 80% of the phosphopeptide motifs peaked at a different time point from their cognate protein (Supplementary Fig. S7B; examples in Fig. 2H). The light-harvesting complex linker protein CP29 (ostta01g04940) illustrates one pattern: its protein level rises in the light while a phosphopeptide motif is dephosphorylated (Supplementary Fig. S7C). This phosphopeptide motif is located adjacent to a target site of chloroplast kinase STN7 in the homologous CP29 of Arabidopsis (Schönberg *et al.*, 2017).

To test the phospho-dawn pattern by a different method, we estimated the bulk protein phosphorylation across the diel cycle using protein gel staining (Supplementary Fig. S9A, B). The proportion of phosphorylated proteins was lowest in the daytime and increased during the night to peak at ZT0 (Supplementary Fig. S9C). The pattern of total phosphorylation estimated by this simpler analysis was therefore broadly consistent with the distribution of phosphopeptide motif profiles (Fig. 2C). Taken together, these results indicate that a regulator other than light or protein abundance controls the *O. tauri* phosphoproteome before dawn. Below, we report phosphosite sequences that suggested its identity.

### Functions of proteins with rhythmic phosphomotifs

**Fig. 4.**
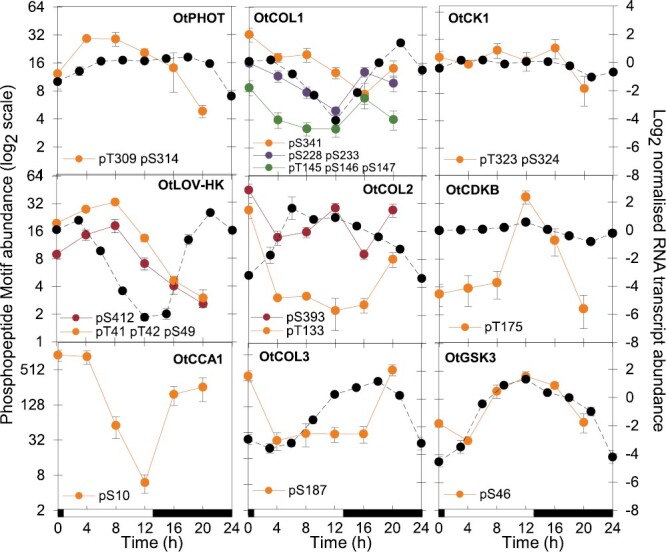
Protein and phosphopeptide motif regulation. Phosphomotif (coloured lines) and RNA profiles (Monnier *et al.*, 2010) (dashed lines) of the photoreceptors, clock components, transcription factors. and kinases indicated, under LD cycles. Left axis range 2^6^ (64-fold) except OtCCA1 (phosphopeptide motif changes 150-fold) and OtCOL2 (phosphopeptide motifs change up to 20-fold). Right (RNA) axis range 12, for log2 data (2^12^=4096-fold in untransformed data). Error bars, SE. Light/dark indicated by white/black bars. PHOT, phototropin photoreceptor; LOV-HK, LOV domain-histidine kinase photoreceptor; COL, CONSTANS-like transcription factor.

The LD datasets confirmed that protein phosphorylation profiles often diverged from protein abundance. The largest cluster PM1 reflected the profiles that peaked in the ZT0 time point (Supplementary Fig. S4B), which also stood out in the PCA (Fig. 1H). Cluster PM1 included 518 phosphopeptide motifs on 395 proteins, and was enriched for GO terms related to transcription, glucose metabolism, K^+^ and protein transport, and ubiquitin-dependent proteolysis functions (similar to PC1 and ZT0-peaking profiles; Supplementary Tables S3–S5). Phosphopeptide enrichment allowed the detection of regulatory proteins including phosphopeptide motifs on predicted CONSTANS-like B-box transcription factors (OtCOLs) related to the plant clock protein TOC1 (Fig. 4), and on the RWP-RK mating-type factor ostta02g04300 (Blanc-Mathieu *et al.*, 2017). PM1 also includes the predicted CK2 target site pS10 in the clock protein CCA1 (ostta06g02340; Fig. 4), close to the homologous location of a CK2 site in Arabidopsis CCA1 (Lu *et al.*, 2011).

Phosphopeptide motifs in cluster PM3 peaked in the light, as expected if their profile was driven by light-stimulated translation of the cognate protein (examples in Fig. 2G). Phosphopeptide motifs on the photoreceptors phototropin and LOV-HK illustrate these daytime profiles (Fig. 4). Protein functions predicted to regulate transcription, metal ion transport, and protein phosphorylation are enriched in this cluster (summarized in Supplementary Fig. S4D; Supplementary Table S4), in profiles with daytime peaks (Supplementary Fig. S5B; Supplementary Table S5), and, along with translation, in profiles contributing to PC2 (Supplementary Table S3).

In contrast, the PM2, PM4, PM7, and PM8 clusters peaked at ZT16, with or without accumulation in daytime (Supplementary Fig. S4B). These clusters are enriched for phosphopeptide motifs on protein kinases including cell cycle-related kinases (Supplementary Figs S4D, S5B; Supplementary Tables S4, S5). Phosphopeptide motif profiles that contributed to PC2 with negative coefficients, related to ZT16 and ZT 20 time points (Fig. 1H), were also enriched for mitosis GO terms, along with Ca^2+^ transmembrane transport (Supplementary Table S3). Consistent with this, terms for mitotic processes (DNA replication and repair) were enriched among dusk-expressed transcripts. We therefore analysed the phospho-regulators that might control these phosphopeptide motif profiles, including potential contributions to non-transcriptional timing.

### Phase-specific target sites

**Fig. 5.**
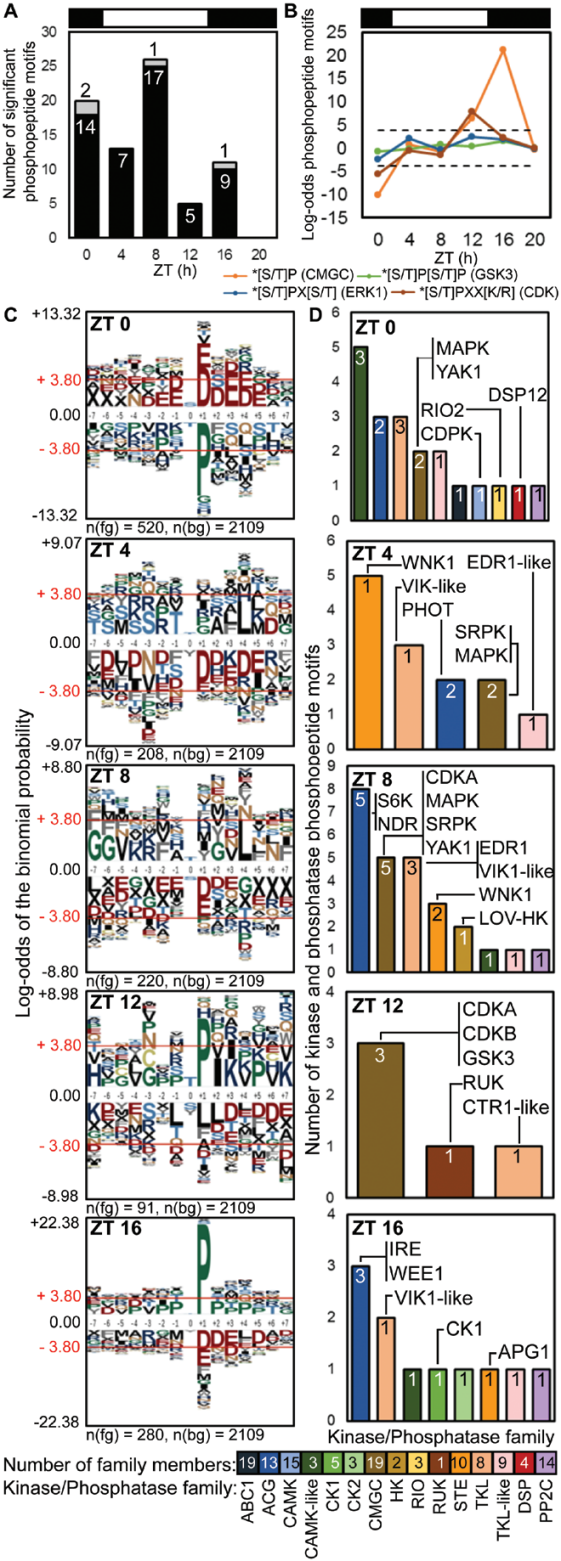
Motif enrichment and rhythmic protein kinases and phosphatases under LD cycles. (A) Rhythmic phosphopeptide motifs peaking at each time point on protein kinase (black) and phosphatase (grey) proteins (numbers). (B) Enrichment of proline-directed motifs, for kinases shown in the key (dashed line, *P*-value=0.05). (C) pLogo sequence motifs of rhythmic phosphopeptide motifs peaking at each time point (foreground; fg), relative to all detected phosphopeptides (background; bg). ±3.80 indicates *P*-value=0.05; residues above and below the axis are over- and under-represented, respectively. (D) Rhythmic phosphopeptide motifs by kinase/phosphatase family, annotated with example proteins.

We first analysed motifs of amino acids that were enriched in rhythmic phosphopeptide motifs, compared with all quantified phosphopeptides to avoid potential detection bias due to phosphopeptide motif abundance. Phosphopeptide motifs that peaked at ZT16 were strikingly enriched for the proline-directed motif [pS/pT]P (Fig. 5B, C). This strongly implicates the CMGC family of protein kinases, including cyclin-dependent kinases (CDKs) and GSK. Consistent with this, the profiles of phosphopeptide motifs with predicted GSK target sequences also most often peaked at ZT16 (Supplementary Fig. S10A). Levels of *GSK3* RNA and a phosphopeptide motif on GSK3 peaked at ZT12 (Fig. 4), though the autophosphorylation site pY210 was not rhythmic (Supplementary Table S2). More specific CDK target motifs [pS/pT]PXX[K/R] were enriched at ZT12 (Fig. 5B), consistent with the known timing of cell division (Farinas *et al.*, 2006; Moulager *et al.*, 2007) and the peak level of the activation phospho-site of CDKB (Fig. 4, centre-right panel). During the day (ZT4 and 8), enrichment of hydrophobic residues at positions –5 and +4 (Fig. 5C) is suggestive of the SnRK consensus (Vlad *et al.*, 2008), the plant kinase most related to animal AMPK.

In contrast, acid([D/E])-directed target motifs were significantly enriched among the rhythmic phosphopeptide motifs that peaked at ZT0, and the proline-directed motifs were depleted (Fig. 5C). Conversely, these acid-directed motifs were depleted on phosphopeptide motifs peaking at ZT16 or ZT4, suggesting a strong phase specificity. Considering the more specific, predicted target sites for the clock-related protein kinases (Supplementary Fig. S10A), more rhythmic phosphopeptide motifs included predicted CK1 targets than CK2 or GSK3 targets, and the phosphorylation of CK1 targets most often peaked at ZT0. Predicted CK2 target sequences had even more phase-specific phosphorylation, with at least 5-fold more peaking at ZT0 than at other times (Supplementary Fig. S10A). Thus predicted targets of the clock-related kinases CK1 and CK2 both contribute to the phospho-dawn profiles, in antiphase to the evening peaks of proline-directed phospho-sites.

### Rhythmic regulation of the kinome

The protein abundance of the three detected protein kinases and two phosphatases was not rhythmic (Supplementary Table S1). We therefore analysed the 68 rhythmic phosphopeptide motifs on protein kinases and five phosphopeptide motifs on protein phosphatases, as candidate mediators of rhythmic phosphorylation (Fig. 5A, D). The phosphopeptide motifs on kinases represent 8% of the total, though protein kinase genes comprise ~1.5% of the genome. Indeed, the most heavily phosphorylated protein with 14 phosphopeptide motifs was the WITH NO LYSINE (WNK) kinase that might target clock proteins in Arabidopsis (Murakami-Kojima *et al.*, 2002) (Supplementary Table S2; Supplementary Fig. S10C). The most changing phosphopeptide motif on a predicted protein phosphatase was pT175 in ostta11g02830, related to human dual-specificity phosphatase DUSP12 (Fig. 5D).

Among the clock-related protein kinases, we noted the dusk-peaking phosphopeptide motif of GSK3 (Fig. 4). CK2 subunits were not detected in our data and the phosphopeptide motif on CK1 was not strongly rhythmic (Fig. 4). A further 21 protein kinases bore rhythmic phosphopeptide motifs that are predicted targets of these clock-related kinases (Supplementary Fig. S10C).

Around mitosis at ZT12–16, significantly peaking phosphopeptide motifs were detected on cell cycle regulators CDKA, CDKB, and WEE1 (Fig. 5D). Kinase phosphopeptide motifs peaking at ZT4–8 included serine-arginine protein kinases (SRPKs), MAPKs, CDKA, and a site on yet another kinase (YAK1). Phosphopeptide motifs that peaked at ZT0, coincident with the phospho-dawn, included RIO2, YAK1, and CDPK, all implicated in cell cycle regulation and progression (Garrett *et al.*, 1991; LaRonde-LeBlanc and Wlodawer, 2005). RIOs are among the few kinase families shared with the Archaea (Kennelly, 2014), making them candidate contributors to an ancient, non-transcriptional oscillator (Edgar *et al.*, 2012).

## Discussion

### The diel proteome and phosphoproteome

Our results contribute to understanding the ‘reactive’ and ‘anticipatory’ components of protein regulation in the green lineage under diel (LD) cycles (Mehta *et al.*, 2021). A small fraction of the *O. tauri* proteins quantified here were rhythmic (just under 10%), compared with a majority (58%) of the phosphopeptide motifs. Of the rhythmic protein profiles, 85% peaked in daytime, consistent with a ‘reactive’ effect due to the light-regulated translation in this organism (Martin *et al.*, 2012), and with enrichment of translation-related functions among daytime-peaking proteins. This result reinforces the dangers of using RNA profiles as a proxy for biological function in general. In this case, however, translation, ribosome biogenesis, and RNA processing functions were enriched among dawn-expressed RNAs (Supplementary Table S5), preceding the enrichment of both translation and chlorophyll biosynthesis GO terms among day-peaking, rhythmic proteins (Supplementary Fig. S5). Observing the expected effects of light-regulated translation further supports our prediction that ‘translational coincidence’ should alter the *O. tauri* proteome in different day-lengths, as some rhythmic RNAs will coincide with light-stimulated translation only in long days (Seaton *et al.*, 2018). Overall, rhythmic proteins in our dataset also have a higher calculated cost of protein expression than non-rhythmic proteins (Laloum and Robinson-Rechavi, 2022), consistent with the notion that rhythmicity might give a selective advantage by limiting this costly protein synthesis to a fraction of the diel cycle.

In contrast, the largest number of phosphopeptide motif profiles peaked in the pre-dawn ZT0 time point. This pattern was consistent with the distribution of the rhythmic phosphopeptide profiles that Kay *et al.* (2021) detected without specific enrichment, which also peaked most often in their pre-dawn interval. The anticipatory ‘phospho-dawn’ might be controlled by the circadian clock. Circadian regulation would be expected to persist under constant conditions, which were not tested here. Studies in Arabidopsis under constant light, however, identified a high fraction of rhythmic phosphopeptides that peaked at subjective dawn (Choudhary *et al.*, 2015; Krahmer *et al.*, 2022), suggesting a similar, circadian-regulated phospho-dawn in higher plants. Such phospho-regulation might prepare green cells for daytime functions and/or end night-time activities, before light-stimulated translation facilitates new protein synthesis.

Acid-directed target sites were clearly enriched at ZT0, implicating the clock-related kinases CK1 and CK2 in regulating the phospho-dawn in *O. tauri*. Enrichment of proline-directed target sites occurs in antiphase, at ZT12–16, which implicates the 19 CMGC-class kinase proteins (Hindle *et al.*, 2014) including CDKs, MAPKs, and GSK3. These phase-specific enrichments were clearer than in the Arabidopsis studies, suggesting that the minimal kinase target network of *O. tauri* might be easier to resolve in the future. Comparison with the specific rhythmic kinases in animals is limited, because the most rhythmic kinase Akt (also known as protein kinase B) in mouse liver (Robles *et al.*, 2017) is absent from the green lineage (Hindle *et al.*, 2014). Rhythmic phosphopeptide targets of CDK1 and CK1D peaked in phosphorylation at a similar time in liver, in the mid night (active) interval (Robles *et al.*, 2017), contrasting with their opposite phases in *O. tauri*. Nonetheless, both the liver and synaptic phosphoproteomes were more rhythmic than the cognate proteomes and showed a different distribution of peak phases (Robles *et al.*, 2017; Brüning *et al.*, 2019), indicating distinctive phospho-regulation. Clusters of peak phosphorylation anticipated the rest–activity transitions in the mouse, consistent with the ‘phospho-dawn’ observed here, in the synaptic phosphoproteome (Brüning *et al.*, 2019) but not in liver (Robles *et al.*, 2017).

The low overall rhythmicity (<10%) in the partial proteome quantified here is consistent with similar studies in Arabidopsis, which identified 0.1–1.5% rhythmic proteins from 7–9 % of the proteome in LD cycles, using iTRAQ labelling with similar statistical criteria to ours (Baerenfaller *et al.*, 2012, 2015), or 4–7% rhythmic proteins from 4% of the proteome under constant light using a gel-based approach (Choudhary *et al.*, 2016). Our results provide 11% coverage in the minimal *O. tauri* proteome, with a more straightforward experimental protocol. Broader coverage of this proteome was reported (Kay *et al.*, 2021) after our preprint was released (Noordally *et al.*, 2018, Preprint), in experiments that included an extensive, high pH reverse phase fractionation, among several technical differences. Their higher reported fraction of rhythmic proteins might reflect the detection of low abundance proteins and/or analysis with no minimum amplitude threshold.

### The ‘dark state’ is indirectly associated with lipid synthesis

Among the rhythmic proteins reported here, some of the most highly regulated were four prasinophyte-specific sequences (unnamed proteins ostta02g03680, ostta03g04960, ostta07g00470, and ostta09g00670; Fig. 3A) along with ostta03g04500 (Supplementary Figs S2A, S8B). These proteins accumulated further in prolonged darkness (Fig. 3A). We previously showed that *O. tauri* stop transcription and cell division in those conditions (O’Neill *et al.*, 2011). Cultures resume gene expression and growth upon return to LD cycles, suggesting that dark adaptation induces a state of cellular quiescence. The ecological relevance of a quiescent ‘dark state’ for photo-autotrophic, surface-dwelling *O. tauri* might not be immediately obvious. However, *Ostreococcus* relatives can persist under the polar night (Joli *et al.*, 2017). Quiescent forms in other phytoplankton (Roy *et al.*, 2014), including in soil or sediments, can be ecologically important in benthic–pelagic coupling (Marcus and Boero, 1998). Cells near the deep chlorophyll maximum (Cardol *et al.*, 2008) could be moved into the dark, benthic zone by turbulence, to return later via upwelling (Countway and Caron, 2006; Collado-Fabbri *et al.*, 2011). Understanding the laboratory ‘dark state’ is therefore likely to have ecological relevance.

Protein content dropped significantly between 12 h and 36 h of darkness (ZT24–ZT48) but was then stable. Cultures of *Chlamydomonas reinhardtii* showed a 50% reduction in protein per cell within 24 h of nitrogen starvation due to a final cell division (Schmollinger *et al.*, 2014), whereas increased cell number did not explain the lower protein content in our dark-adapting cultures. Proteins associated with cytosolic translation were notably depleted (Supplementary Table S6), rather than abundant chloroplast proteins involved in photosynthesis. Photosynthetic functions might be particularly important to recover from quiescence, similar to the rapid regrowth observed after nutrient starvation (Liefer *et al.*, 2018).

Our culture conditions included sorbitol and glycerol in the growth medium, which are required for viability in prolonged darkness (O’Neill *et al.*, 2011) and can probably support metabolic activity in *O. tauri* (Smallwood *et al.*, 2018b, Preprint). Both dark-accumulating and dark-depleted proteins identified in our studies overlapped with proteins that were similarly regulated under nitrogen depletion, particularly when combined with increased carbon availability (Smallwood *et al.*, 2018a, Preprint). Nitrogen depletion is commonly used to induce lipid synthesis in algae, in the context of third-generation biofuel production (Zienkiewicz *et al.*, 2016). Both chloroplast and ribosomal proteins can be depleted in these conditions, though only a subset of lipid metabolic proteins accumulate (Schmollinger *et al.*, 2014). Prolonged darkness and/or hypoxia can also induce lipid accumulation, and hypoxia can occur in dark-adapting algal cultures due to continued respiration (Hemschemeier *et al.*, 2013). Our ‘dark state’ proteome might therefore reflect active lipid synthesis from the sorbitol and glycerol in the growth medium.

*Ostreococcus tauri* can form both intracellular lipid droplets and extracellular droplets in membrane-bound ‘pea-pod’ structures (Smallwood *et al.*, 2018b, Preprint). Lipid droplets in other algae include major proteins that are restricted to limited taxonomic groups (Zienkiewicz *et al.*, 2016), so functionally equivalent proteins in *O. tauri* might be specific to the prasinophyte group. Some lipid droplet proteins are predicted to have all-α-helical structure, including the major lipid droplet protein Cre09.g405500 of *C. reinhardtii* or the lipid droplet surface protein (LDSP) of the stramenophile *Nannochloropsis oceanica*. Protein structure homology modelling aligned ostta02g03680 with a human BAR domain dimer, an all-helical protein domain that can sense and create membrane curvature (Simunovic *et al.*, 2015) (Supplementary Fig. S11), suggesting that this *O. tauri* protein might also be involved in lipid droplets. *Nannochloropsis oceanica* lipid synthesis and LDSP accumulation is highly rhythmic but day-phased (Poliner *et al.*, 2015). The night-expressed proteins in *O. tauri* indirectly suggest a different regulation of lipid synthesis, that could have biotechnological relevance. Future studies to understand the transition to the ‘dark state’ in *O. tauri* will need to consider both cellular metabolite pools and extruded components, such as lipid droplets.

## Supplementary data

The following supplementary data are available at *JXB* online.

Fig. S1. Identification of outlier phosphopeptide replicate 4E.

Fig. S2. Most detected protein and phosphopeptide motif profiles, with comprehensive heat maps, clusters, and enriched functions.

Fig. S3. Changing phosphopeptide motifs on non-changing proteins.

Fig. S4. Clustered protein and phosphopeptide motif profiles with examples.

Fig. S5. GO enrichments for peaks and troughs.

Fig. S6. Simulation of light-regulated translation.

Fig. S7. Loci identified in both LD protein and phosphopeptide motif datasets.

Fig. S8. Regulation of proteins tested under dark adaptation.

Fig. S9. Protein and phosphoprotein abundance in an LD cycle.

Fig. S10. CK1, CK2, and GSK3 kinase targets and phosphorylation sites in rhythmic kinases.

Fig. S11. Structural homology of a rhythmic prasinophyte-specific protein.

Table S1. Proteins quantified under LD cycles.

Table S2. Phosphopeptide motifs quantified under LD cycles.

Table S3. GO term enrichment among RNA, proteins, and phosphopeptide motifs contributing to PCA.

Table S4. GO term enrichment among RNA, proteins, and phosphopeptide motifs in clusters.

Table S5. GO term enrichment among rhythmic proteins and phosphopeptide motifs by peak/trough times.

Table S6. Proteins quantified under dark adaptation.

erad290_suppl_supplementary_figures_S1-S11

erad290_suppl_supplementary_table_S1

erad290_suppl_supplementary_table_S2

erad290_suppl_supplementary_table_S3

erad290_suppl_supplementary_table_S4

erad290_suppl_supplementary_table_S5

erad290_suppl_supplementary_table_S6

## Data Availability

The OTTH95 strain is available from the CCAP (www.ccap.ac.uk) and RCC (roscoff-culture-collection.org) stock centres. Mass spectrometry proteomics data have been deposited in the ProteomeXchange Consortium via the PRIDE partner repository with the dataset identifiers: LD global proteomics, PXD001735; LD phosphoproteomics, PXD001734; DA global proteomics, PXD002909. The LC-MS data were also previously available in pep2pro at www.pep2pro.ethz.ch (Assemblies ‘*Ostreococcus tauri* Light:dark cycle, LD global’, ‘*Ostreococcus tauri* Light:dark cycle, LD phospho’, ‘*Ostreococcus tauri* dark adaptation, DA global’). Processed data lists are provided in the supplementary data, and are publicly available from the Zenodo repository https://doi.org/10.5281/zenodo.7742118. (Hindle *et al.*, 2023).

## Abbreviations

CCA1: circadian clock associated 1 protein
CK1: casein kinase 1
CK2: casein kinase 2
CMGC: cyclin-dependent kinase, mitogen-activated protein kinase, glycogen synthase kinase, CDC-like kinase
DA: dark adaptation
GSK3: glycogen synthase kinase 3
LD: light–dark
PC: principal component
PCA: principal component analysis
ZT: Zeitgeber time
