# Supplementary material for: A phospho-dawn of protein modification anticipates light onset in the picoeukaryote Ostreococcus tauri
Source: J Exp Bot. 2023 Jul 22;74(18):5514–31. doi: 10.1093/jxb/erad290 (PMC10540734; doi:10.1093/jxb/erad290)
Supplement: erad290_suppl_supplementary_figures_S1-S11 [file erad290_suppl_supplementary_figures_s1-s11.pdf]

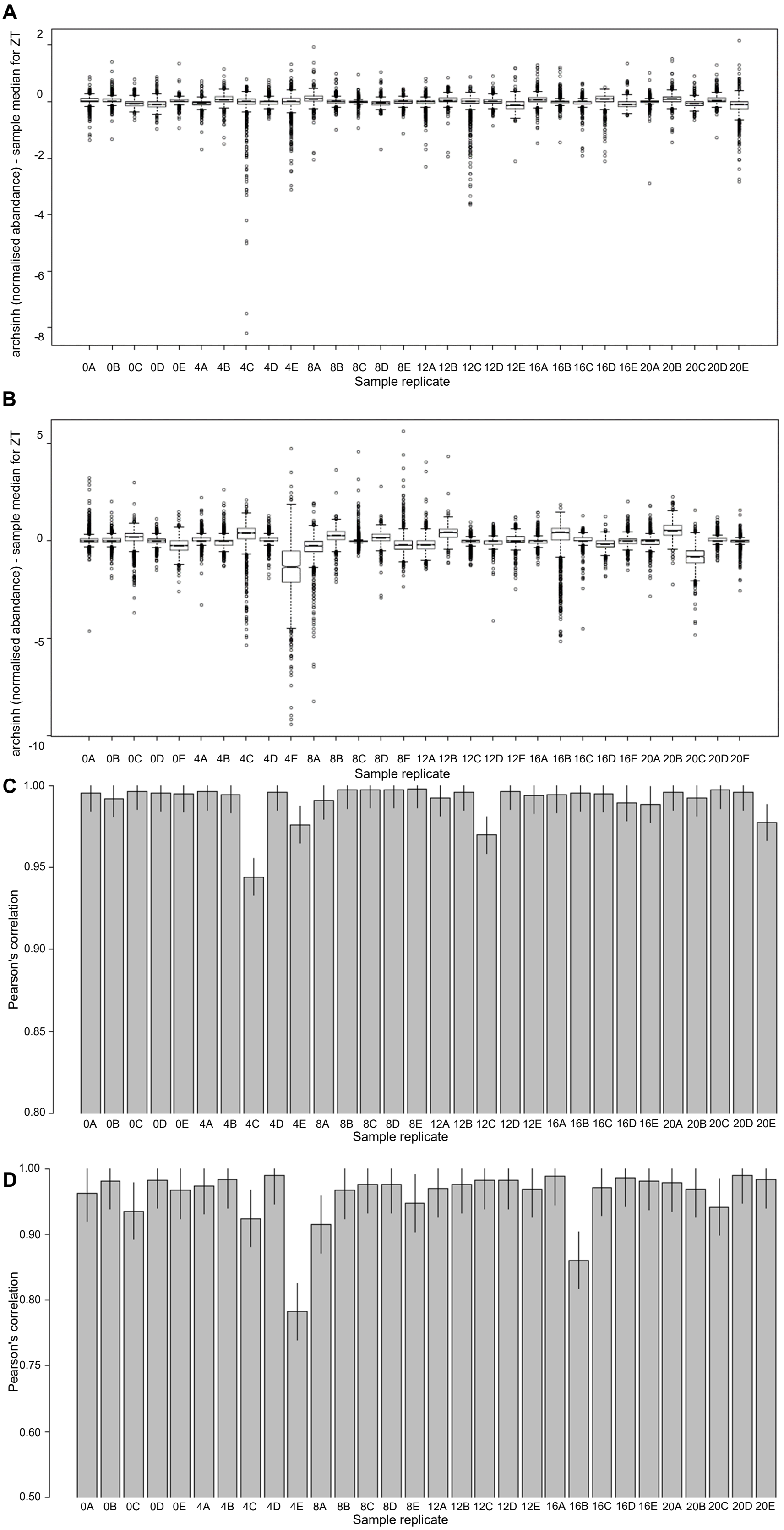

A
archsinh (normalised abandance) - sample median for ZT
2
0
-2
-4
-6
-8
0A 0B 0C 0D 0E 4A 4B 4C 4D 4E 8A 8B 8C 8D 8E 12A 12B 12C 12D 12E 16A 16B 16C 16D 16E 20A 20B 20C 20D 20E
Sample replicate
B
archsinh (normalised abandance) - sample median for ZT
5
0
-5
-10
0A 0B 0C 0D 0E 4A 4B 4C 4D 4E 8A 8B 8C 8D 8E 12A 12B 12C 12D 12E 16A 16B 16C 16D 16E 20A 20B 20C 20D 20E
Sample replicate
C
Pearson's correlation
1.00
0.95
0.90
0.85
0.80
0A 0B 0C 0D 0E 4A 4B 4C 4D 4E 8A 8B 8C 8D 8E 12A 12B 12C 12D 12E 16A 16B 16C 16D 16E 20A 20B 20C 20D 20E
Sample replicate
D
Pearson's correlation
1.00
0.90
0.80
0.75
0.60
0.50
0A 0B 0C 0D 0E 4A 4B 4C 4D 4E 8A 8B 8C 8D 8E 12A 12B 12C 12D 12E 16A 16B 16C 16D 16E 20A 20B 20C 20D 20E
Sample replicate

Supplementary Figure S1. Identification of outlier phosphopeptide replicate 4E. Pearson's correlation for (A) proteins and (B) phosphopeptide motifs and sample replicate r^2 respective to median abundance at a ZT for (C) proteins and (D) phosphopeptide motifs. Note differing scales in (A,B), (C,D).

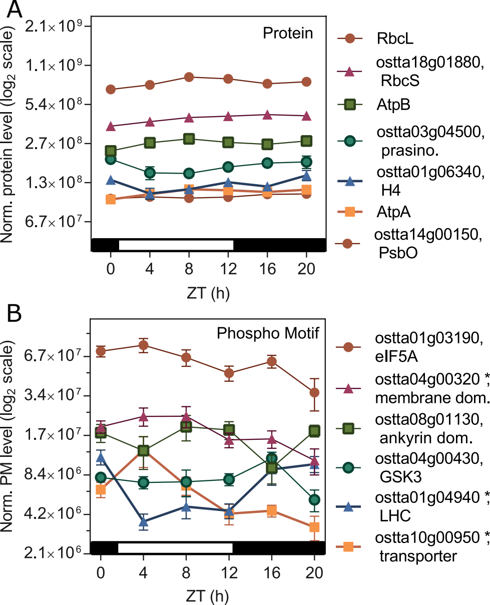
A
Protein
Norm. protein level (log2 scale)
2.1×10^9
1.1×10^9
5.4×10^8
2.7×10^8
1.3×10^8
6.7×10^7
0
4
8
12
16
20
ZT (h)
RbcL
ostta18g01880, RbcS
AtpB
ostta03g04500, prasino.
ostta01g06340, H4
AtpA
ostta14g00150, PsbO
B
Phospho Motif
Norm. PM level (log2 scale)
6.7×10^7
3.4×10^7
1.7×10^7
8.4×10^6
4.2×10^6
2.1×10^6
0
4
8
12
16
20
ZT (h)
ostta01g03190, eIF5A
ostta04g00320 *, membrane dom.
ostta08g01130, ankyrin dom.
ostta04g00430, GSK3
ostta01g04940 *, LHC
ostta10g00950 *, transporter

Supplementary Figure S2. Most-detected protein and PM profiles. with comprehensive heat maps, clusters and enriched functions. Highly-abundant proteins (A) and PMs (B) under LD conditions (* marks rhythmic PMs). Error bars, S.E. Light/dark indicated by white/black bars, above.

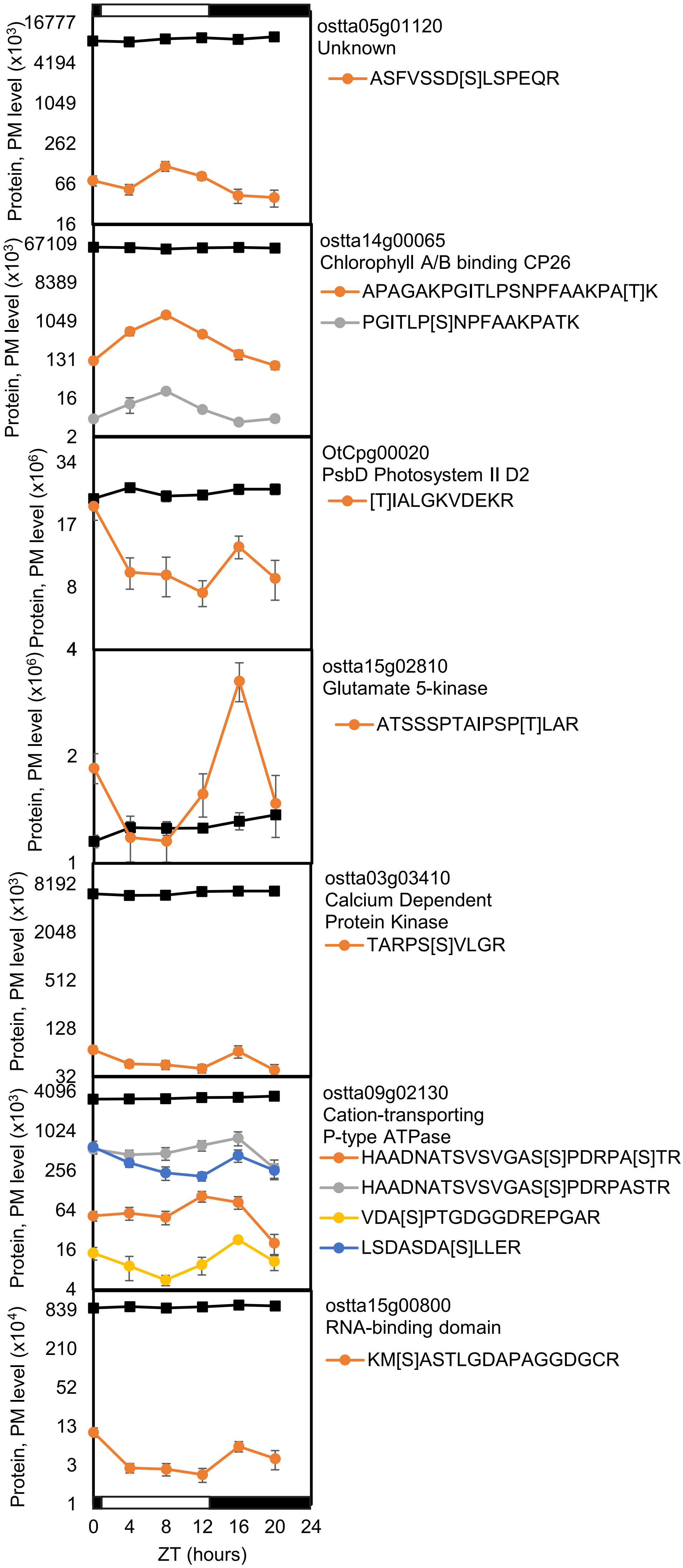

ostta05g01120
Unknown
ASFVSSD[S]LSPEQR
ostta14g00065
Chlorophyll A/B binding CP26
APAGAKPGITLPSNPFAAKPA[T]K
PGITLP[S]NPFAAKPATK
OtCpg00020
PsbD Photosystem II D2
[T]IALGKVDEKR
ostta15g02810
Glutamate 5-kinase
ATSSSPTAIPSP[T]LAR
ostta03g03410
Calcium Dependent
Protein Kinase
TARPS[S]VLGR
ostta09g02130
Cation-transporting
P-type ATPase
HAADNATSVSVGAS[S]PDRPA[S]TR
HAADNATSVSVGAS[S]PDRPASTR
VDA[S]PTGDGGDREPGAR
LSDASDA[S]LLER
ostta15g00800
RNA-binding domain
KM[S]ASTLGDAPAGGDGCR
Protein, PM level (x10³)
Protein, PM level (x10⁶)
Protein, PM level (x10⁴)
16777
4194
1049
262
66
16
67109
8389
1049
131
16
2
34
17
8
4
2
1
8192
2048
512
128
32
4096
1024
256
64
16
4
839
210
52
13
3
1
0
4
8
12
16
20
24
ZT (hours)

Supplementary Figure S3. Changing PMs on non-changing proteins. Significantly non-changing proteins (Black lines) determined by two one-sided tests (TOST; $\varepsilon = 0.3$), plotted with their rhythmic phosphopeptide motifs ± S.E., square brackets show phosphorylated residue. Light/dark indicated by white/black bars.

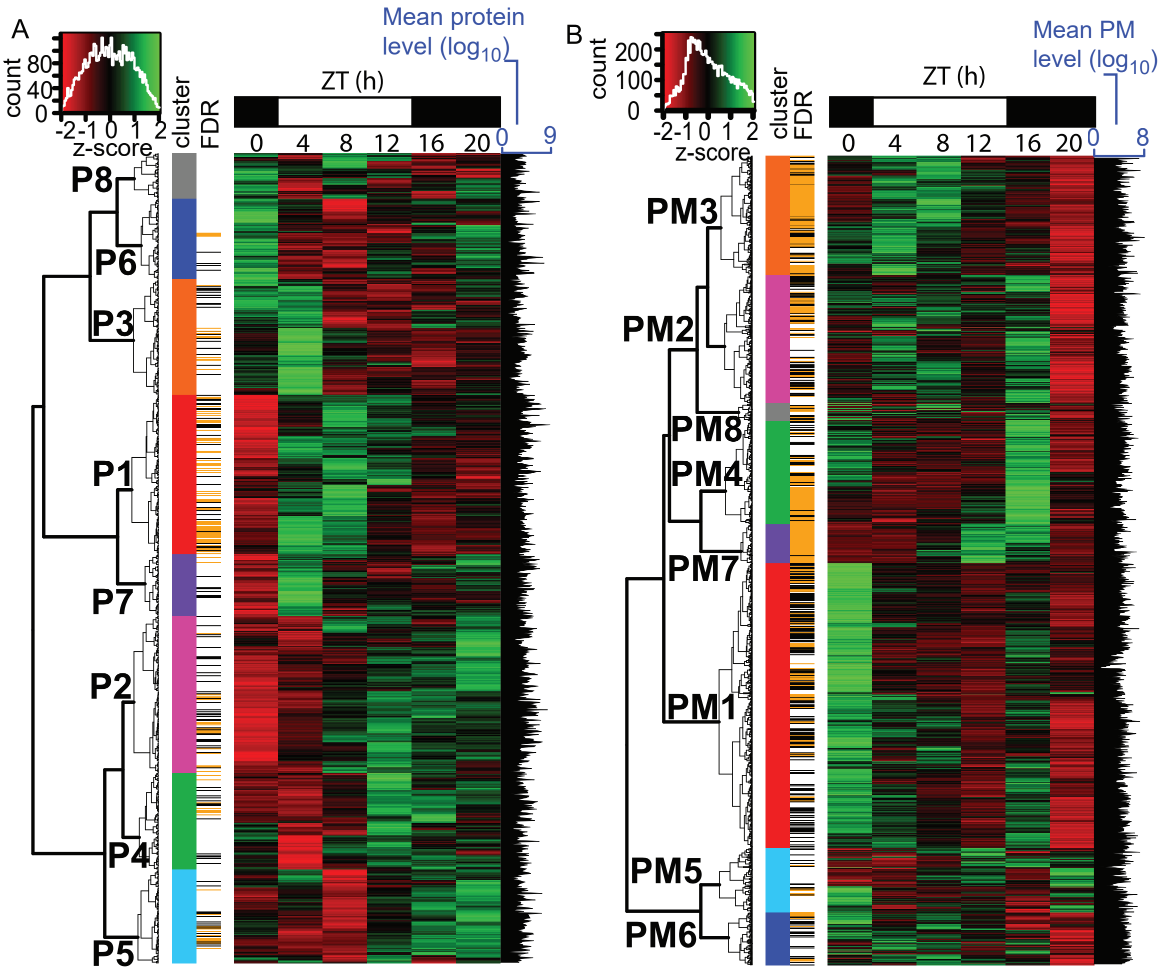

C **PROTEIN CLUSTERS**

P1 Ribosomal subunits, translation initiation/elongation
P2 Thioredoxin-like, FNR, PGK, GAPDH, RuBisCO, PPDK, malate dehydrogenase CBM20, PLP transferase
P3 Chlorophyll A/B binding, PSII PsbM
P4 PLP transferases, SSII
P5 Thioredoxin-like, SQR/FR cytochrome b5, phosphogluconate dehydrogenase, transaldolase, aldehyde dehydrogenase
P6 Prasinophyte-specific proteins, NH_4+ transporter
P7 Cytochrome b6, ribosomal subunits, translation initiation/elongation

D **PHOSPHOPEPTIDE MOTIF CLUSTERS**

PM1 TFs, ZF, Myb; cold-shock protein; Zn, Na/solute, Mg2+, transport; AGC, YAK1, CAMK-like, RIO2, CDPK; PIPK; PsaM **PM2** TFs, ZF; eIFs; MAPK,APG1, NDR, AGC, CAMK-like; PP2C;6-phosphofructo-2-kinase; Ca ATPase **PM3** TFs, ZFs; eIFs; chlorophyll A/B binding, RubisCO activase; thioredoxin; K, Mg, ABC transport; FHA; AGC, SRPK, CDKA, CDKC ; PIPK **PM4** TFs ZF; PEP carboxylase proteosome; NO_3^- , Mg transport; WEE1, IRE, MAPK; PIPK **PM5** TFs, CO; GAPDH;chlorophyll A/B binding; Asn synthetase; T6P synthase; **PM6** eIFs, ribosome biogenesis; S6K; PIPK, diacylglycerol kinase
PM7 CDKA, CDKB, GSK3; PPDK; phospholipid ATPase
PM8 WNK1, EDR1, CDK regulatory subunit

Supplementary Figure S4. Clustered protein and PM profiles with examples. Heat maps of median-normalised (A) protein and (B) PM abundance, with insets top left showing the distribution of levels and colour scale. Clusters P1-8 or PM1-8 are shown, colours in 'cluster' track are as in Figure 1D-1I; FDR track shows >1.5 fold-change and BH FDR adjusted *p*-value <0.05 (black line) or <0.01 (orange line); bars to right of each panel show the mean protein or PM abundance (log_{10} scale). Light/dark indicated by white/black bars, above. (C, D) Examples of significantly-changing proteins and PMs in each cluster (as noted in the main text).

a Protein - Biological Process

ZT	GO.ID	Term	Annotated	Significant	Expected	*p*-value
0	GO:0098662	inorganic cation transmembrane transport	17	1	0.1	9.40E-02
	GO:0006412	translation	116	17	6.8	1.30E-04
4	GO:0006412	translation	116	16	4.82	1.30E-06
	GO:0006414	translational elongation	8	2	0.33	4.00E-02
	GO:0006260	DNA replication	3	3	0.04	1.40E-06
8	GO:0015995	chlorophyll biosynthetic process	7	4	0.24	3.10E-05
	GO:0006414	translational elongation	8	2	0.27	2.70E-02
	GO:0006412	translation	116	9	3.95	3.00E-02
	GO:0006418	tRNA aminoacylation for protein translation	15	1	0.03	2.80E-02
12	GO:0006260	DNA replication	3	3	0.07	1.20E-05
	GO:1901606	alpha-amino acid catabolic process	5	2	0.12	4.50E-02
	GO:0090150	establishment of protein localization to membrane	4	1	0.02	2.30E-02
20	GO:0015995	chlorophyll biosynthetic process	7	1	0.04	3.90E-02
	GO:0006414	translational elongation	8	1	0.05	4.50E-02

b Phosphopeptide motifs - Biological Process

ZT	GO.ID	Term	Annotated	Significant	Expected	*p*-value
0	GO:0035556	intracellular signal transduction	17	6	3.38	3.10E-03
	GO:0046488	phosphatidylinositol metabolic process	6	4	1.19	8.10E-03
	GO:0006457	protein folding	4	3	0.79	1.55E-02
	GO:0006413	translational initiation	4	3	0.79	1.55E-02
	GO:0006468	protein phosphorylation	48	14	9.53	1.62E-02
	GO:0050896	response to stimulus	34	11	6.75	2.49E-02
	GO:0034622	cellular macromolecular complex assembly	5	3	0.99	2.69E-02
	GO:0055085	transmembrane transport	38	12	7.55	3.23E-02
	GO:0015031	protein transport	22	7	4.37	3.29E-02
	GO:0006352	DNA-templated transcription, initiation	5	3	0.99	3.42E-02
	GO:0006468	protein phosphorylation	48	5	1.17	2.60E-03
4	GO:0055085	transmembrane transport	38	11	3.97	1.20E-05
	GO:0006355	regulation of transcription, DNA-templated	22	8	2.3	7.50E-05
	GO:0000160	phosphorelay signal transduction system	6	3	0.63	6.70E-03
	GO:0008152	metabolic process	231	29	24.15	1.20E-02
	GO:0072525	pyridine-containing compound biosynthetic process	3	2	0.31	1.57E-02
	GO:0044271	cellular nitrogen compound biosynthetic process	46	12	4.81	2.07E-02
	GO:0009108	coenzyme biosynthetic process	5	2	0.52	4.74E-02
	GO:0006355	regulation of transcription, DNA-templated	22	6	1.3	6.10E-04
	GO:0009168	purine ribonucleoside monophosphate biosynthetic proc	3	2	0.18	8.20E-03
	GO:0009152	purine ribonucleotide biosynthetic process	4	2	0.24	1.58E-02
	GO:0046129	purine ribonucleoside biosynthetic process	4	2	0.24	1.58E-02
	GO:0034654	nucleobase-containing compound biosynthetic process	40	10	2.37	4.11E-02
	GO:0006813	potassium ion transport	7	2	0.41	5.01E-02
8	GO:0006468	protein phosphorylation	48	18	4.93	8.40E-10
	GO:0032012	regulation of ARF protein signal transduction	3	2	0.31	1.80E-02
	GO:0005992	trehalose biosynthetic process	3	2	0.31	1.80E-02
	GO:0006355	regulation of transcription, DNA-templated	22	5	2.26	2.50E-02
	GO:0006511	ubiquitin-dependent protein catabolic process	3	2	0.13	4.10E-03
	GO:0007017	microtubule-based process	4	2	0.17	8.00E-03
	GO:1901362	organic cyclic compound biosynthetic process	46	3	2	2.01E-02
	GO:0044238	primary metabolic process	192	12	8.36	2.87E-02
	GO:0098662	inorganic cation transmembrane transport	9	2	0.39	4.29E-02
12	GO:0006261	DNA-dependent DNA replication	3	2	0.11	3.10E-03
	GO:0006468	protein phosphorylation	48	5	1.84	1.58E-02
	GO:0006396	RNA processing	7	2	0.27	1.98E-02
	GO:0006974	cellular response to DNA damage stimulus	10	2	0.38	3.19E-02
	GO:0019219	regulation of nucleobase-containing compound metabolic process	23	2	0.88	3.26E-02
	GO:0006457	protein folding	4	3	0.28	8.70E-04
	GO:0050896	response to stimulus	34	6	2.37	2.99E-02
	GO:0008152	metabolic process	231	27	16.1	4.15E-02
16	GO:0006281	DNA repair	9	5	1.08	8.20E-04
	GO:0006310	DNA recombination	4	3	0.48	3.65E-03
	GO:0009168	purine ribonucleoside monophosphate biosynthetic process	3	2	0.36	2.82E-02
	GO:0006468	protein phosphorylation	48	9	5.77	4.15E-02
	GO:0006355	regulation of transcription, DNA-templated	22	3	0.5	8.60E-03
	GO:0007165	signal transduction	19	2	0.43	3.91E-02
20	GO:0006412	translation	11	1	0.02	1.90E-02
	GO:0006468	protein phosphorylation	48	28	16.64	2.80E-07
	GO:0055085	transmembrane transport	38	20	13.17	7.50E-05
	GO:0006355	regulation of transcription, DNA-templated	22	13	7.63	5.60E-04
	GO:0008152	metabolic process	231	105	80.09	8.70E-04
	GO:0016310	phosphorylation	52	32	18.03	2.27E-03
	GO:0000160	phosphorelay signal transduction system	6	5	2.08	4.50E-03
	GO:0046488	phosphatidylinositol metabolic process	6	5	2.08	4.50E-03
	GO:0009058	biosynthetic process	83	45	28.78	8.60E-03
	GO:0006796	phosphate-containing compound metabolic process	76	42	26.35	9.00E-03
	GO:0044249	cellular biosynthetic process	76	40	26.35	9.53E-03
	GO:0035556	intracellular signal transduction	17	13	5.89	1.31E-02
	GO:0006812	cation transport	22	11	7.63	1.45E-02
	GO:0006352	DNA-templated transcription, initiation	5	4	1.73	1.54E-02
	GO:0009108	coenzyme biosynthetic process	5	4	1.73	1.54E-02
	GO:0072525	pyridine-containing compound biosynthetic process	3	3	1.04	1.55E-02
	GO:0032012	regulation of ARF protein signal transduction	3	3	1.04	1.55E-02
	GO:0030258	lipid modification	3	3	1.04	1.55E-02
	GO:0090407	organophosphate biosynthetic process	9	4	3.12	1.57E-02
	GO:0044711	single-organism biosynthetic process	28	14	9.71	4.57E-02

Supplementary Figure S5. GO enrichments for peaks and troughs. GO Biological Process term enrichment for rhythmic (A) proteins and (B) phosphopeptide motifs, that was significant (Fisher's exact test p-value <0.05) in profiles with peak (no shading) or trough (pink shading) time at each timepoint. Light/dark indicated by white/black column. Grey bars represent proportion of significant terms identified with respect to total number of background annotated terms.

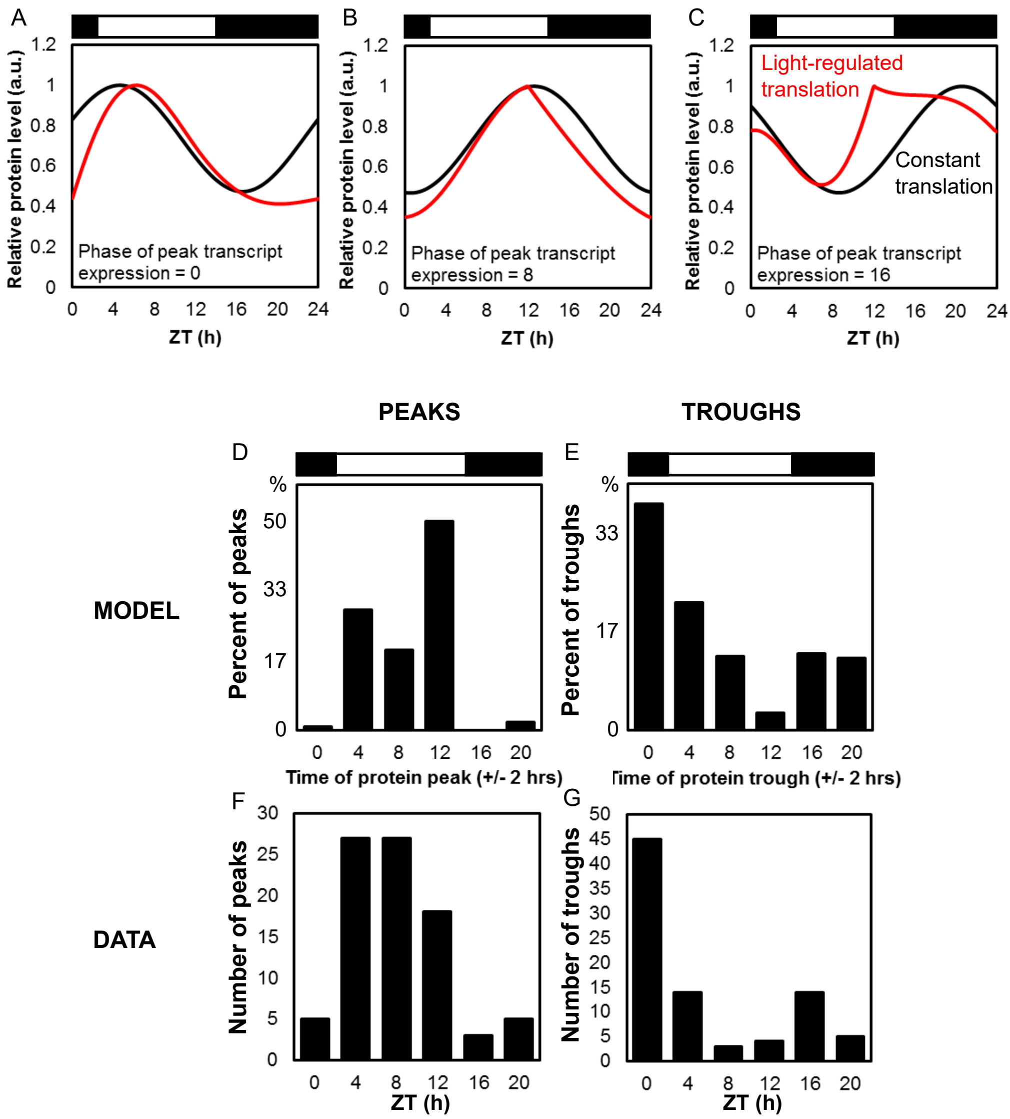
A
B
C
Relative protein level (a.u.)
1.2
1
0.8
0.6
0.4
0.2
0
Phase of peak transcript expression = 0
Phase of peak transcript expression = 8
Phase of peak transcript expression = 16
Light-regulated translation
Constant translation
0 4 8 12 16 20 24
ZT (h)
PEAKS
TROUGHS
MODEL
DATA
D
E
F
G
%
Percent of peaks
Percent of troughs
50
33
17
0
Time of protein peak (+/- 2 hrs)
Time of protein trough (+/- 2 hrs)
Number of peaks
Number of troughs
30 25 20 15 10 5 0
50 45 40 35 30 25 20 15 10 5 0
0 4 8 12 16 20
ZT (h)

Supplementary Figure S6. Simulation of light-regulated translation. (A-C) Simulation of protein dynamics for an RNA with peak expression at ZT0 (A), ZT8 (B) and ZT16 (C), with observed, light-regulated translation rate (red lines) or with constant translation rate (black lines). Distribution of protein peaks (D,F) and troughs (E,G) for the model with light-regulated translation (D,E) compared to data (F,G). Distributions for constant translation would reflect the distribution of RNA profiles.

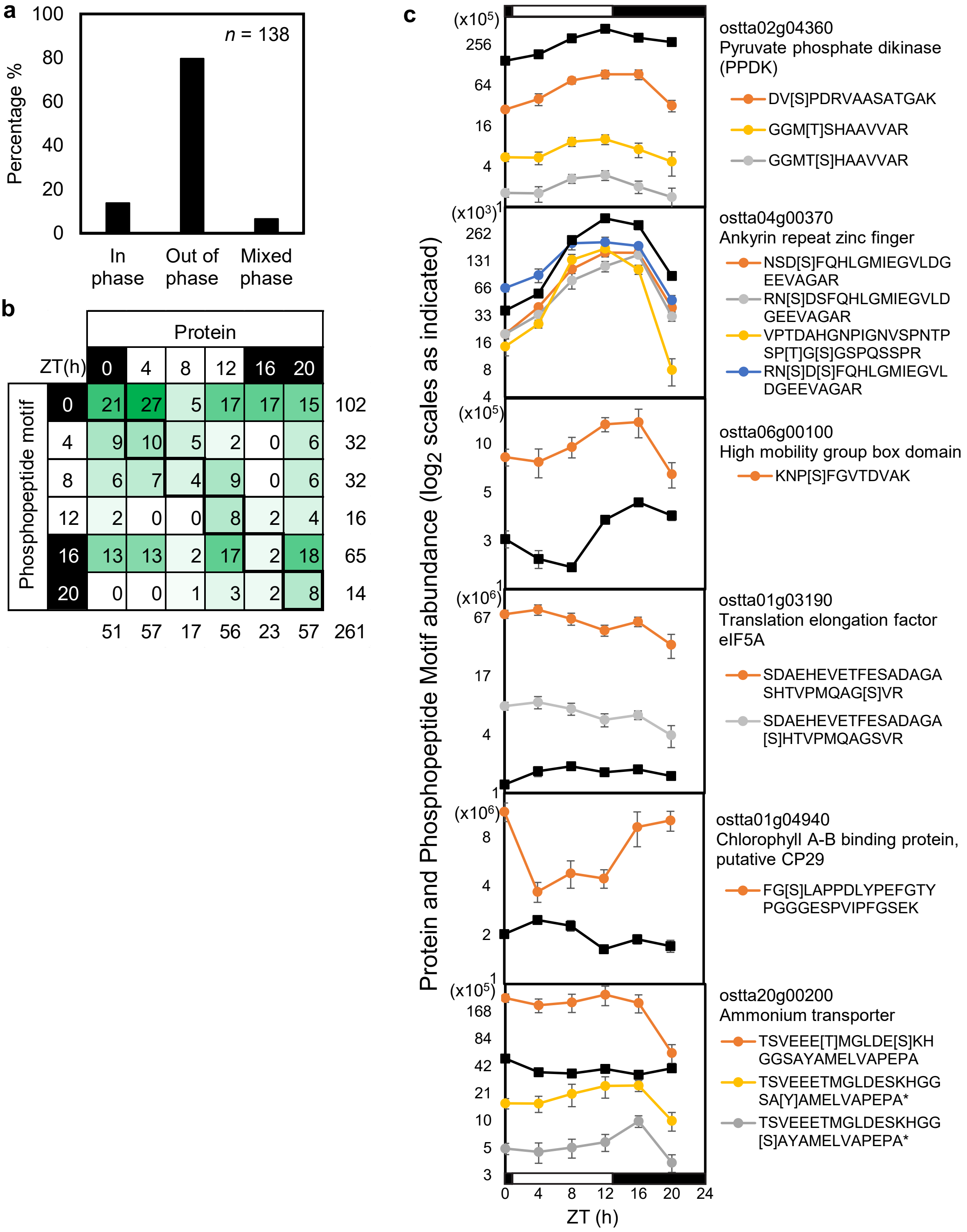
a
Percentage %
100
80
60
40
20
0
n = 138
In phase
Out of phase
Mixed phase
b
Protein
ZT(h)
0 4 8 12 16 20
Phosphopeptide motif
0 | 21 27 5 17 17 15 | 102
4 | 9 10 5 2 0 6 | 32
8 | 6 7 4 9 0 6 | 32
12 | 2 0 0 8 2 4 | 16
16 | 13 13 2 17 2 18 | 65
20 | 0 0 1 3 2 8 | 14
51 57 17 56 23 57 261
c
Protein and Phosphopeptide Motif abundance (log2 scales as indicated)
(x10⁵)
256
64
16
4
ostta02g04360
Pyruvate phosphate dikinase (PPDK)
DV[S]PDRVAASATGAK
GGM[T]SHAAVVAR
GGMT[S]HAAVVAR
(x10³)
262
131
66
33
16
8
4
ostta04g00370
Ankyrin repeat zinc finger
NSD[S]FQHLGMIEGVLDG EEVAGAR
RN[S]DSFQHLGMIEGVLD GEEVAGAR
VPTDAHGNPIGNVSPNTP SP[T]G[S]GSPQSSPR
RN[S]D[S]FQHLGMIEGVL DGEEVAGAR
(x10⁵)
10
5
3
ostta06g00100
High mobility group box domain
KNP[S]FGVTDVAK
(x10⁶)
67
17
4
1
ostta01g03190
Translation elongation factor eIF5A
SDAEHEVETFESADAGA SHTVPMQAG[S]VR
SDAEHEVETFESADAGA [S]HTVPMQAGSVR
(x10⁶)
8
4
2
ostta01g04940
Chlorophyll A-B binding protein, putative CP29
FG[S]LAPPDLYPEFGTY PGGGESPVIPFGSEK
(x10⁵)
168
84
42
21
10
5
3
ostta20g00200
Ammonium transporter
TSVEEE[T]MGLDE[S]KH GGSAYAMELVAPEPA
TSVEEETMGLDESKHGG SA[Y]AMELVAPEPA*
TSVEEETMGLDESKHGG [S]AYAMELVAPEPA*
0 4 8 12 16 20 24
ZT (h)

Supplementary Figure S7. Loci identified in both LD protein and phosphopeptide motif datasets. (A, B) Peak time is compared for genes identified in both LD protein and phosphopeptide motif datasets, with examples (C). (A) Mixed phase: multiple PMs, peaking at same and different times from cognate protein. Green shading in (B) follows number per bin. Plotting conventions in (C) follow Figure 2G, 2H.

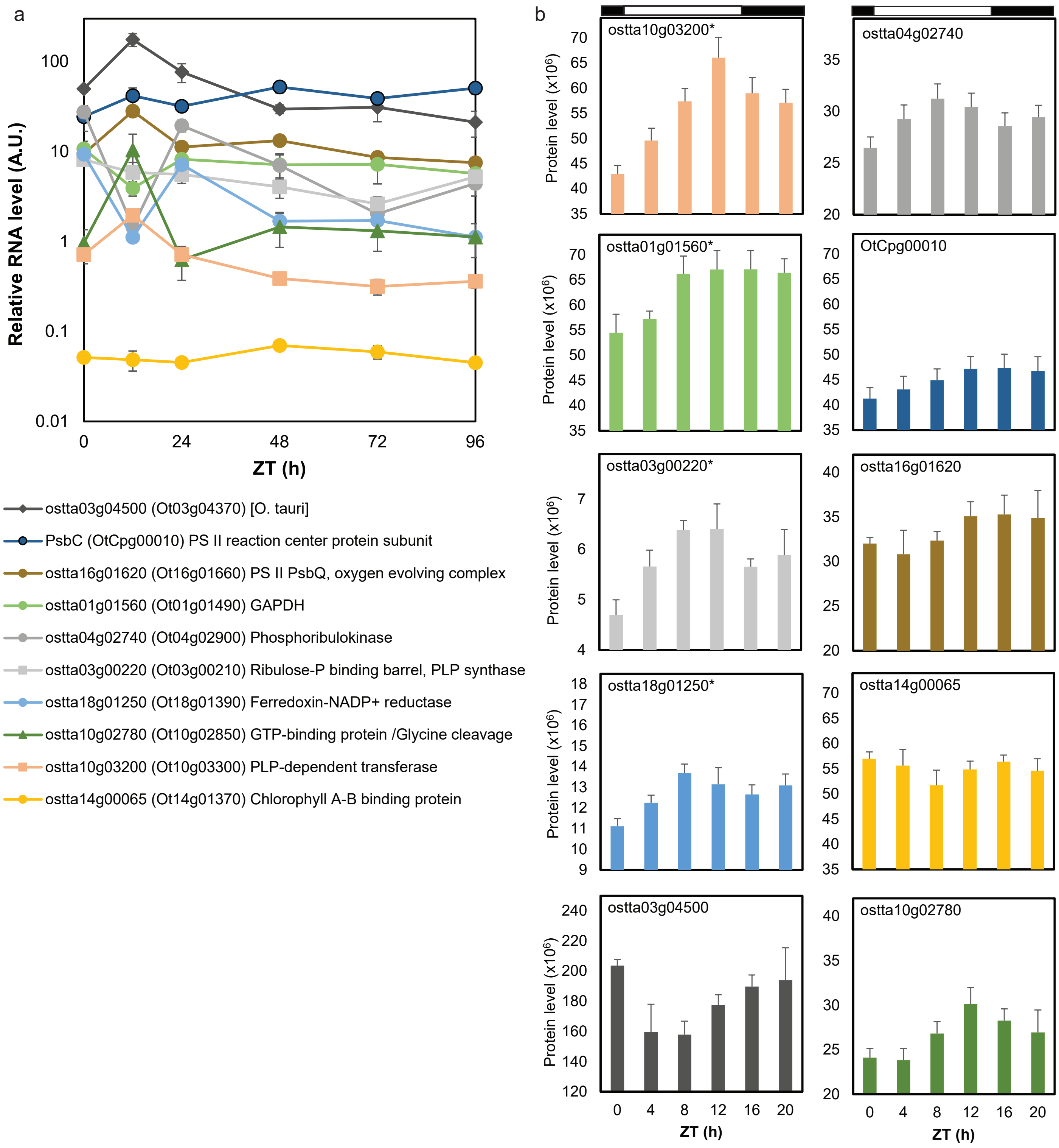
a
Relative RNA level (A.U.)
100
10
1
0.1
0.01
0
24
48
72
96
ZT (h)
ostta03g04500 (Ot03g04370) [O. tauri]
PsbC (OtCpg00010) PS II reaction center protein subunit
ostta16g01620 (Ot16g01660) PS II PsbQ, oxygen evolving complex
ostta01g01560 (Ot01g01490) GAPDH
ostta04g02740 (Ot04g02900) Phosphoribulokinase
ostta03g00220 (Ot03g00210) Ribulose-P binding barrel, PLP synthase
ostta18g01250 (Ot18g01390) Ferredoxin-NADP+ reductase
ostta10g02780 (Ot10g02850) GTP-binding protein /Glycine cleavage
ostta10g03200 (Ot10g03300) PLP-dependent transferase
ostta14g00065 (Ot14g01370) Chlorophyll A-B binding protein
b
ostta10g03200*
ostta04g02740
ostta01g01560*
OtCpg00010
ostta03g00220*
ostta16g01620
ostta18g01250*
ostta14g00065
ostta03g04500
ostta10g02780
Protein level (x10^6)
0
4
8
12
16
20
ZT (h)

Supplementary Figure S8. Regulation of proteins tested under Dark Adaptation (DA).
For ten proteins compared in the DA and metabolic labelling (Martin *et al.*, 2012) data (Figure 3C), (A) RNA abundance under LD and DA conditions from qRT-PCR assays, and (B) protein profiles under LD. *, rhythmic proteins. Error bar, S.E.

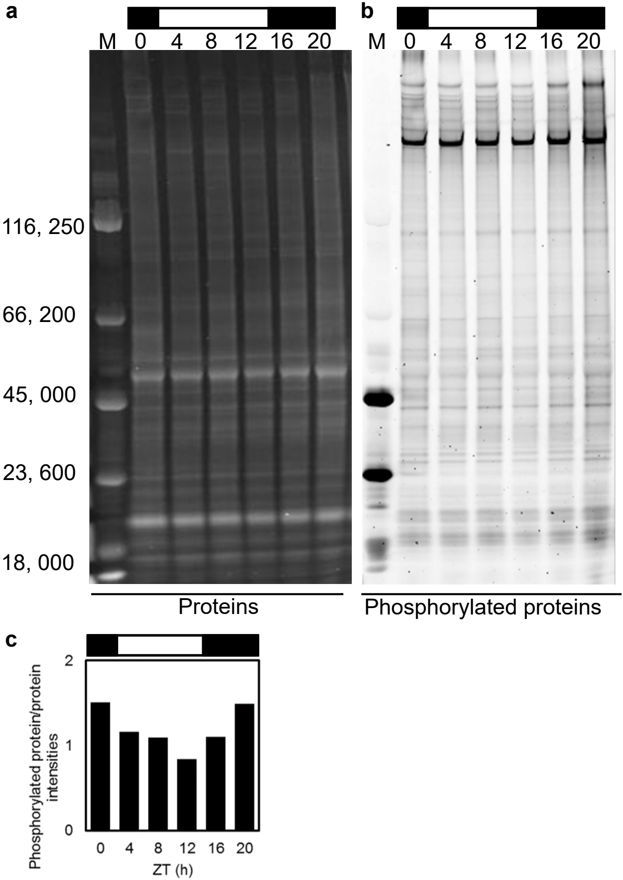
a
M 0 4 8 12 16 20
116, 250
66, 200
45, 000
23, 600
18, 000
Proteins
b
M 0 4 8 12 16 20
Phosphorylated proteins
c
Phosphorylated protein/protein intensities
2
1
0
0 4 8 12 16 20
ZT (h)

Supplementary Figure S9. Protein and phospho-protein abundance in LD cycle. Stained gels showing changes in (A) protein and (B) phosphorylated protein abundance in LD, with (C) ratio of quantified, phosphorylated protein to total protein intensity.

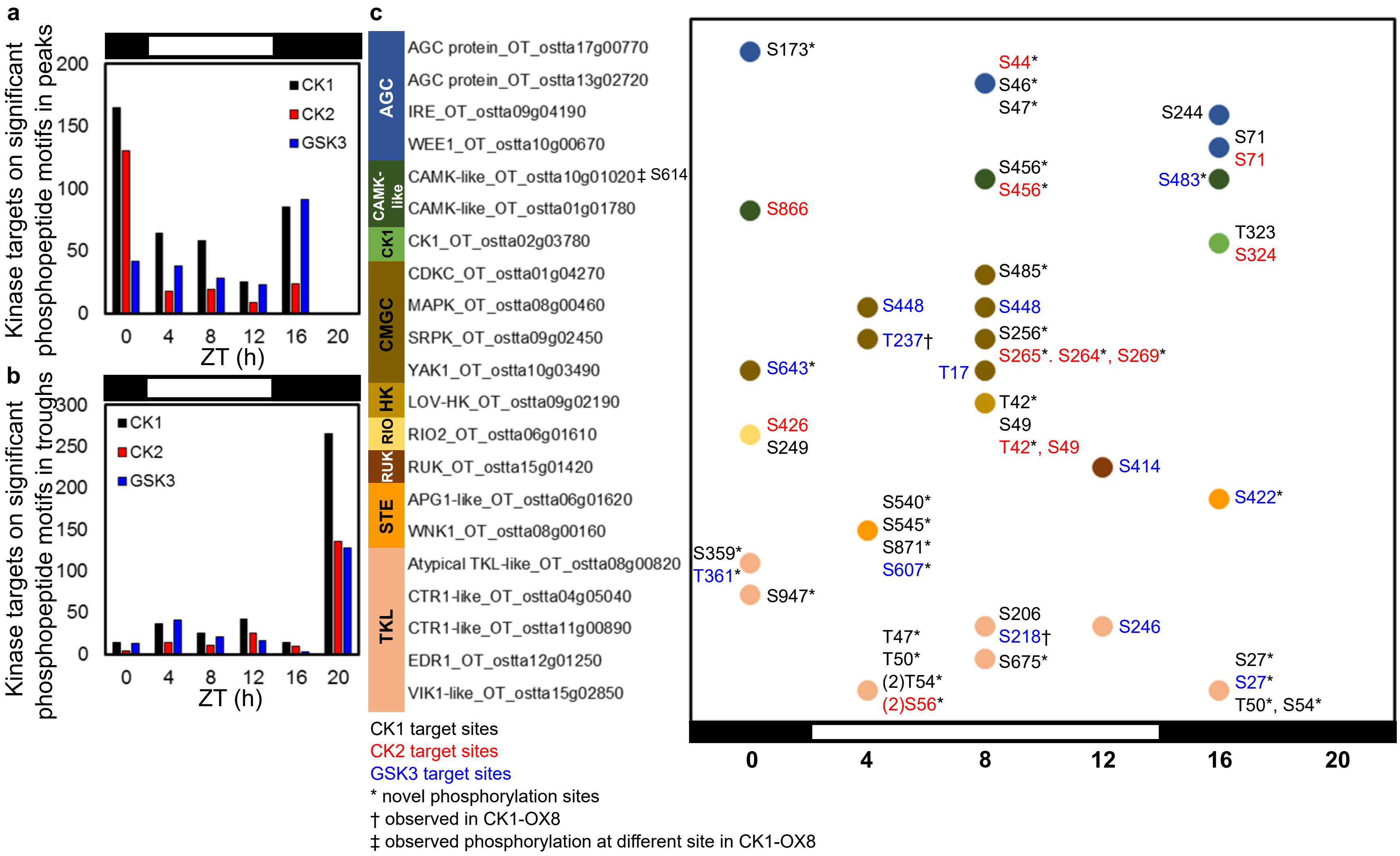

a
Kinase targets on significant phosphopeptide motifs in peaks
CK1
CK2
GSK3
0
50
100
150
200
0 4 8 12 16 20
ZT (h)
b
Kinase targets on significant phosphopeptide motifs in troughs
CK1
CK2
GSK3
0
50
100
150
200
250
300
0 4 8 12 16 20
ZT (h)
c
AGC
CAMK-like
CK1
CMGC
HK
RIO
RUK
STE
TKL
AGC protein_OT_ostta17g00770
AGC protein_OT_ostta13g02720
IRE_OT_ostta09g04190
WEE1_OT_ostta10g00670
CAMK-like_OT_ostta10g01020‡ S614
CAMK-like_OT_ostta01g01780
CK1_OT_ostta02g03780
CDKC_OT_ostta01g04270
MAPK_OT_ostta08g00460
SRPK_OT_ostta09g02450
YAK1_OT_ostta10g03490
LOV-HK_OT_ostta09g02190
RIO2_OT_ostta06g01610
RUK_OT_ostta15g01420
APG1-like_OT_ostta06g01620
WNK1_OT_ostta08g00160
Atypical TKL-like_OT_ostta08g00820
CTR1-like_OT_ostta04g05040
CTR1-like_OT_ostta11g00890
EDR1_OT_ostta12g01250
VIK1-like_OT_ostta15g02850
S173*
S44*
S46*
S47*
S244
S71
S71
S456*
S456*
S483*
S866
T323
S324
S485*
S448
S448
T237†
S256*
S265*. S264*, S269*
S643*
T17
T42*
S49
T42*, S49
S426
S249
S414
S422*
S540*
S545*
S871*
S607*
S359*
T361*
S947*
S206
S218†
S246
T47*
T50*
S675*
(2)T54*
(2)S56*
S27*
S27*
T50*, S54*
0 4 8 12 16 20
CK1 target sites
CK2 target sites
GSK3 target sites
* novel phosphorylation sites
† observed in CK1-OX8
‡ observed phosphorylation at different site in CK1-OX8

Supplementary Figure S10. CK1, CK2 and GSK3 kinase targets and phosphorylation sites in rhythmic kinases. Distribution of GPS3-predicted CK1 (black), CK2 (red) and GSK3 (blue) targets among rhythmic phosphopeptide motifs, binned by peak (A) and trough (B) times. (C) Phosphosites on rhythmic protein kinases predicted to be phosphorylated by CK1, CK2 and GSK3, site location labels coloured as in (A). * sites first reported here; †‡ sites observed previously (van Ooijen *et al.*, 2013). Protein kinase classes are coloured as in Figure 5.

A

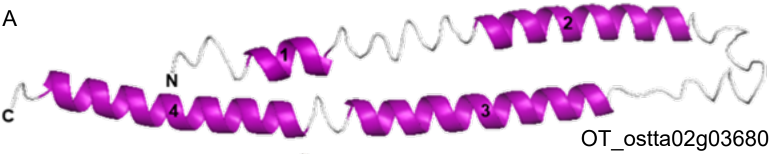
N
1
2
C
4
3
OT_ostta02g03680

B

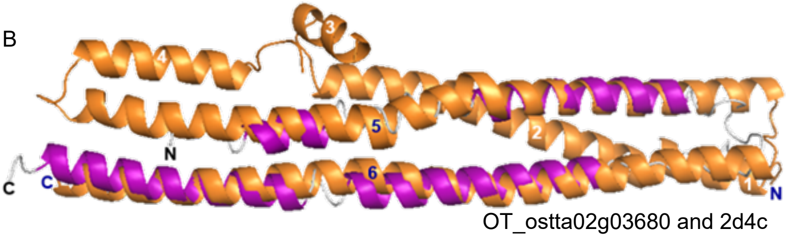
3
4
5
2
N
C
C
6
1
N
OT_ostta02g03680 and 2d4c

Supplementary Figure S11. Structural homology of a rhythmic prasinophyte-specific protein. Structural homology models predicted using I-TASSER of (A) ostta02g03680 where the model is overlaid with (B) *H. sapiens* BAR domain structure (2d4c). Model α-helices (purple) and β-sheets (green) are numbered in black on the *O. tauri* model and in blue where structure is conserved with homologue protein overlay and in white where secondary structure is not conserved.
